# Effect of General Practitioner Training in a Collaborative Child Mental Health Care Program on Children’s Mental Health Outcomes in a Low-Resource Setting: A Cluster Randomized Trial

**DOI:** 10.1001/jamapsychiatry.2022.3989

**Published:** 2022-11-30

**Authors:** Vandad Sharifi, Zahra Shahrivar, Hadi Zarafshan, Sheida Beiky Ashezary, Fariba Arabgol, Mojgan Khademi, Morteza Jafarinia, Ahmad Hajebi, Farid Abolhassani, Soheila Emami, Ali Beiki Ashkezari, Elizabeth A. Stuart, Ramin Mojtabai, Lawrence Wissow

**Affiliations:** 1 Department of Psychiatry, Roozbeh Hospital, Tehran University of Medical Sciences, Tehran, Iran; 2 Psychiatry and Psychology Research Center, Roozbeh Hospital, Tehran University of Medical Sciences, Tehran, Iran; 3 Department of Child and Adolescent Psychiatry, Emam Hosain Hospital, Tehran, Iran; 4 Department of Psychiatry, Imam Hossein Hospital, Shahid Beheshti University of Medical Sciences, Tehran, Iran; 5 Monash University, Melbourne, Victoria, Australia; 6 Mildura Base Public Hospital, Mildura, Victoria, Australia; 7 Research Center for Addiction & Risky Behaviors (ReCARB), Iran Psychiatric Hospital, Iran University of Medical Sciences, Tehran, Iran; 8 National Institute of Health Research, Tehran, Iran; 9 Private practice, Tehran, Iran; 10 School of Industrial Engineering, College of Engineering, University of Tehran, Tehran, Iran; 11 Johns Hopkins Bloomberg School of Public Health, Baltimore, Maryland; 12 University of Washington School of Medicine, Seattle

## Abstract

**Importance:**

Integrated care for children is rarely studied, especially in low- and middle-income countries, where generalists often provide mental health care.

**Objectives:**

To explore the effect of adding a child and youth component to an existing adult collaborative care program on mental health outcomes and receipt of care.

**Design, Setting, and Participants:**

This cluster randomized trial was conducted within an adult collaborative care program in Tehran, Iran. General practitioners (GPs), their 5- to 15-year-old patients, and patients’ parents were included. Children and youths coming for routine medical visits who scored greater than the cutoff on the Strengths and Difficulties Questionnaire (SDQ) were followed up for 6 months. The study was conducted from May 2018 to October 2019, and analysis was conducted from March 2020 to August 2021.

**Interventions:**

GPs were randomized to either a 2.5-day training on managing common child mental health problems (intervention) or refresher training on identification and referral (control).

**Main Outcomes and Measures:**

Primary outcome was change in SDQ total problems score; secondary outcomes included discussion of psychosocial issues by the GPs and receipt of mental health care during the follow-up period.

**Results:**

Overall, 49 GPs cared for 389 children who scored greater than the cutoff on the SDQ (216 children in intervention group, 173 in control group). Patients’ mean (SD) age was 8.9 (2.9) years (range, 5 to 15 years), and 182 (47%) were female patients. At 6 months, children in the intervention group had greater odds of receiving mental health care during the study (odds ratio [OR], 3.0; 95% CI, 1.1 to 7.7), parents were more likely to report that intervention GPs had discussed parent (OR, 2.1; 95% Cl, 1.1 to 3.8) and child (OR, 2.0; 95% Cl, 0.9 to 4.8) psychosocial issues, and intervention GPs were more likely to say they had provided counseling (OR, 1.8; 95% Cl, 1.02 to 3.3). However, there was no greater improvement in SDQ scores among children seen by intervention vs control GPs. Adjusted for clustering within GP, the variables used for balanced allocation (practice size, practice ownership, and study wave), and the other variables associated with change in SDQ scores over time, there was not a significant time-treatment interaction at either the 3- or 6-month follow-up points (linear combination of coefficients for intervention, 0.57 [95% CI, –1.07 to 2.22] and –0.08 [95%CI, –1.76 to 1.56], respectively). In a subgroup of GPs with practices composed of 50% or more children, children seen by intervention GPs improved to a significantly greater extent (–3.6 points; 95% CI, –6.7 to –0.46 points; effect size *d* = 0.66; 95% CI, 0.30 to 1.01) compared with those seen by control GPs.

**Conclusions and Relevance:**

In this cluster randomized trial, GP training on managing common child mental health problems did not demonstrate greater improvement in child SDQ scores. Child mental health training for GPs in collaborative care can improve children’s access to mental health care, but prior experience working with children and their families may be required for GPs to use a brief training in a way that improves child outcomes.

**Trial Registration:**

ClinicalTrials.gov Identifier: NCT03144739

## Key Points

**Question:**

Is it possible to improve child mental health by adding a child and youth component to an adult-focused integrated care program in a middle-income country?

**Findings:**

In this cluster randomized trial with 49 general practitioners (GPs) caring for 389 children and youths, child mental health training for GPs increased children’s receipt of mental health care (GPs more likely to counsel, parents more likely to report that their children had received mental health care) compared with GPs instructed only to refer. Across all GPs, children’s mental health improved similarly regardless of training; however, among GPs whose patients were predominantly children, training significantly improved child mental health status compared with instruction to refer.

**Meaning:**

These findings suggest that it may be feasible to expand adult-focused GP collaborative care models to include children and youth; however, GPs with more experience with children may be best able to take advantage of a brief training that expands their mental health role.

## Teaser

This cluster randomized trial explores the effect of adding a child and youth component to an existing collaborative care program in Iran on child mental health outcomes and receipt of care.

## Introduction

Worldwide, 10% to 20% of children develop mental health problems, but most remain untreated,^1^ especially in low- and middle-income countries (LMICs).^2^ For adults, integrated care has proven to be a way to fill a similar gap,^3^ but there have been few studies among children, most coming from higher-income countries.^4,5^ We conducted a child integrated care trial in Iran, an LMIC whose medical system resembles other countries with a mix of public and private insurance and with general practitioners (GPs) as a gateway to services across the lifespan.^6^

Iran launched adult integrated care in 1989 with a collaborative model linking urban GPs with community mental health centers (CMHCs).^7^ GPs collaboratively manage adult mental health problems but only detect and refer children and youths (ie, patients aged <16 years). We investigated the efficacy of adding a child and youth component to the existing adult program in Tehran. We hypothesized that trained GPs would provide more mental health treatment to children and youths and that children and youths with mental health problems seeing trained GPs would experience greater improvement in symptoms. We planned subgroup analyses to explore variation in outcomes by child, parent, and GP characteristics.^8,9,10,11^

## Methods

### Study Design

The study used a cluster randomized design (children clustered within GPs).^8^ It was approved by the institutional review boards of the Johns Hopkins Medical Institutions and the Tehran University of Medical Sciences. The protocol has been published^8^ and registered (Supplement 1).

### GP Participants

GPs were recruited from the 75 GPs participating in the adult collaborative care program and provided written informed consent. All were eligible unless they saw few or no children. GPs in the program receive periodic training in mental health care and receive incentives if patients complete follow-up visits. GPs are visited by psychologists from the CMHCs to audit and discuss cases. GPs can refer patients to the CMHCs for consultation or ongoing care.

### Child and Parent Participants

Child and youth participants were aged 5 to 15 years, seeking care for any reason from a participating GP. During 2 to 3 weeks for each GP, research assistants systematically approached adults accompanying potentially eligible children (henceforth referred to as *parent*). Children in distress (as determined by the GP or their office staff), unable to speak Farsi, or in active treatment at a participating CMHC were excluded. Consenting parents answered the parent form of the Strengths and Difficulties Questionnaire (SDQ),^12^ an instrument measuring childhood mental health and behavioral problems. Children scoring greater than the Iranian cut points^13^ (total problems score ≥15 for age 5-11 years and ≥17 for age ≥12 years) were enrolled in the trial. Parents consented to provide information about their child, themselves, their interactions with the GP, and other care their child might receive.

### Randomization and Masking

Consenting GPs were randomized to receive either enhanced training as described in the next section (intervention group) or a refresher in identification and referral alone (control group). Intervention GPs were encouraged to treat nonacute child mental health problems if parents and patients were willing; control GPs were instructed to continue referring to a CMHC. All GPs received support from the program as before.

Randomization used the Stata routine ralloc to create a nonfactorial balanced list of allocations in which GPs were assigned in blocks of 2 or 4 to the intervention or control group, such that 2 GP characteristics (practice size [small: <20 patients of all ages/d; large: ≥20 patients of all ages/d] and ownership [private vs public]) were balanced in the intervention and control groups. Practice ownership was hypothesized as important for balancing because privately insured patients might have more access to mental health services outside the collaborative care network,^6^ and practice size was hypothesized to be (inversely) related to the amount of time GPs would be able to spend with individual patients. The balancing process also created allocations across 3 waves of GPs entering the study at intervals of 3 to 4 months. One investigator (L.W.), blinded to GPs’ identities, randomly ordered a list of consenting GPs and matched GPs sequentially to the first open slot on the allocation list that matched their practice characteristics. This yielded each GP’s treatment and wave assignment.

Research assistants assessed outcomes by telephone. Assistants knew which GP the child had seen but were blinded to training assignments. GPs were not given SDQ scorings but knew that it had been administered.

### Procedures

Intervention training was based on past work,^14^ the World Health Organization’s Mental Health Gap,^5^ and information from stakeholders.^15^ Training was designed to help GPs identify problems, engage families, and provide brief interventions including (1) transdiagnostic problem solving and help with parent-child interactions and (2) condition-specific brief treatments. Training used lectures, discussion, and practice with standardized patients over 2.5 days. Control GPs received a 1-day refresher covering problem recognition and description of treatment options available through the CMHCs.

### Primary Outcome

The primary outcome was the parent-rated SDQ total problems score, measured at baseline, 3 months, and 6 months. These time points were selected in recognition of the curvilinear pattern of improvement often seen in studies of child mental health treatment^16^ and to allow maximal use of data should children be lost to follow-up at 6 months. The SDQ is a brief (25-item) tool covering emotions (low mood and anxiety), attention, conduct, and peer interactions. It is sensitive to clinically important change, and its total score represents the mix of problems that children and youths often present in primary care. The Farsi version of the parent SDQ for younger children has been validated against the Farsi Child Behavior Checklist^17^ and the Kiddie Schedule for Affective Disorders and Schizophrenia present and lifetime version.^18^

### Secondary Outcomes

At baseline and 6 months, parents reported on their child’s mental health care in the prior 6 months. Parents’ mental health status, a factor associated with child mental health,^11^ was measured with the 28-item General Health Questionnaire (GHQ)^19^ at baseline, 3 months, and 6 months. The GHQ, previously used in Iran, has good sensitivity and specificity for common mental disorders (cut-point ≥24), and can be used as a continuous measure of distress.^20,21^

Immediately following each child’s enrollment visit, GPs were asked (1) if they believed the child or youth had a behavioral or emotional problem warranting treatment and, (2) if so, what treatment they provided (eg, counseling, medication, referral), if any. Independently, parents reported on whether emotional or behavioral problems (the child’s or their own) had been discussed. We asked both parents and GPs about visit content because, in a prior study, we found that parents’ and doctors’ recall could differ.^22^

### Baseline Child, Parent, and GP Characteristics

Parent and child age and gender, family economic security, and length of relationship with the GP were recorded at baseline. GP characteristics, including gender, length of time at the practice site, prior training in child mental health or development, and proportion of their practice made up by children and youth, were also recorded.

### Statistical Analysis

The power calculation, following methods outlined by Hemming et al,^23^ was based on a plan to recruit at least 45 GPs from the collaborative care network. The first step ignored clustering within GPs and estimated, based on a minimally detectable effect size of approximately *d* = 0.34 and a 2-sided alpha = .05, a power of 0.8,^24,25^ needing 135 patients per study group (270 total patients). The second step adjusted this number for clustering. A review of adult primary care studies from high-income countries found a median intraclass correlation coefficient (ICC) of 0.01 for a general mental health screening measure^26^; 270 patients among 45 GPs yields clusters of 6; an estimated ICC of 0.01 yields a design effect of 1 + ([6 − 1) × 0.01) = 1.05, or a sample size at outcome of approximately 284. Assuming a 20% loss to follow-up would require enrolling 355 patients.

Study groups remained masked until exploratory analyses were completed. Missing items in the SDQ and GHQ were addressed using methods described for the scales.^27,28^ Of 1106 SDQs administered at baseline, less than 1% had 1 or more missing items; all but 3 (<1%) could have total scores imputed.^27^ Of 448 GHQs administered at baseline, 59 (12%) had 1 or more items missing. All but 13 (3%) could be scored by prorating subscale totals.^28^

Analyses were conducted in 3 stages: (1) effect of intervention on change in SDQ scores from baseline to the 3- and 6-month follow-ups; (2) effects of intervention on secondary outcomes, including receipt of mental health care during the study, change in parental mental health, and parent and GP reports of care at the index visit; and (3) planned subgroup analyses corresponding to GP characteristics. We additionally explored possible heterogeneity related to parent and child characteristics (child age, child gender, and parent GHQ status).

As planned, we initially used multilevel regression (child or parent observations clustered within GPs) with the interaction of time (as a categorical variable) and GP group assignment as a predictor of child or parent outcomes.^29,30^ A similar approach, but without a time-group interaction, was used for outcomes that referred only to the enrollment visit. We used random-intercept regression models, included covariates used in the balanced allocation (study wave, practice size, practice ownership),^31^ and fit the model using maximum likelihood with Stata mixed and melogit commands for continuous and binary outcomes, respectively. Stata version 16.1 (StataCorp) was used. eAppendix 1 in Supplement 2 provides details of the planned statistical procedures used in this report, and eAppendix 2 in Supplement 2 demonstrates how alternative approaches to analysis yield conclusions that are similar to the ones derived from the planned analyses.

## Results

### Recruiting and Baseline Characteristics

**Figure 1.**
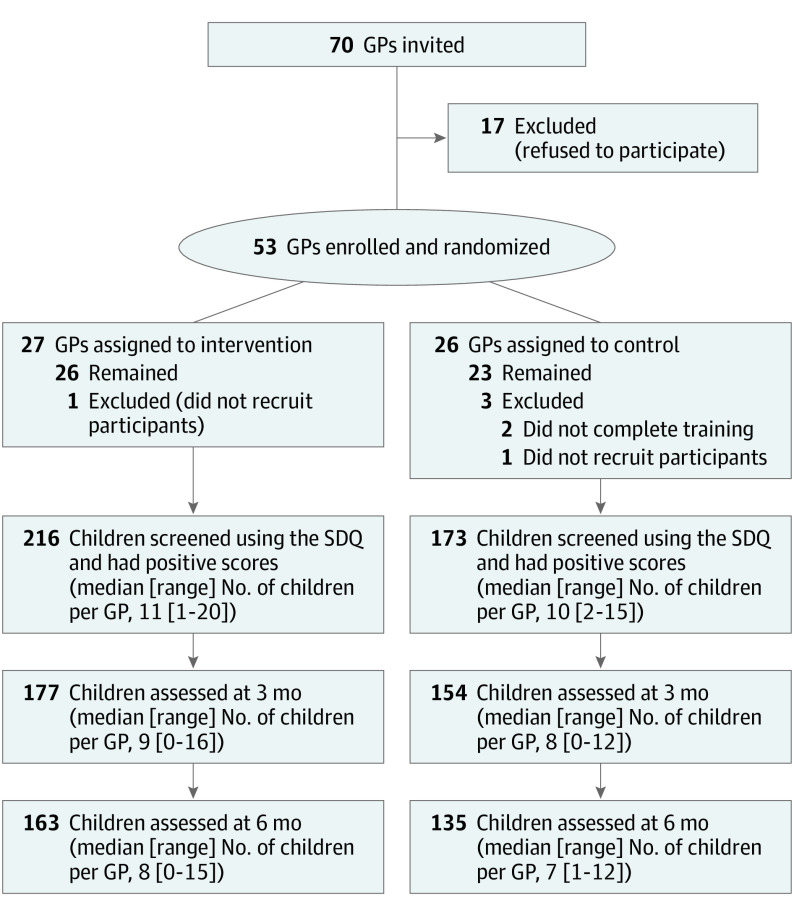
Study Flow Diagram GP indicates general practitioner; SDQ, Strengths and Difficulties Questionnaire.

GP recruiting occurred in January to February 2018. Of 75 GPs in the collaborative, 70 initially indicated interest, and 53 provided consent and were randomized (26 control, 27 intervention). Two GPs who were randomized could not be scheduled for training (both control participants), and for 2 GPs, no eligible patients were recruited (1 each in the intervention and control groups) (Figure 1). Intervention and control GPs were similar in practice characteristics, but intervention GPs were more likely to be female (14 [54%]) compared with control GPs (5 [22%]) (Table 1). All but 2 GPs were in solo practice; the 2 at the same site worked nonoverlapping shifts.

**Table 1.** Baseline Study Participant Characteristics by Observation Time Point

Characteristics	Participants, No. (%)								
	At baseline			At 3 mo			At 6 mo		
	Intervention	Control	Total	Intervention	Control	Total	Intervention	Control	Total
**GPs**									
No.	26	23	49	22	24	46	23	23	46
Female	14 (54)	5 (22)	19 (39)	13 (54)	5 (23)	18 (39)	12 (52)	5 (22)	17 (37)
Private practice^a^	21 (81)	19 (83)	40 (82)	19 (79)	18 (82)	37 (80)	18 (78)	19 (83)	37 (80)
Large practice, ie sees ≥20 patients/d^a^	13 (50)	12 (52)	25 (51)	13 (54)	12 (55)	25 (54)	13 (57)	12 (52)	25 (54)
Predominantly child practice, ie ≥50% of patients are children	8 (32)	11 (48)	19 (40)	7 (30)	10 (45)	17 (38)	7 (32)	11 (48)	18 (40)
At practice site ≥10 y	16 (64)	18 (78)	34 (71)	14 (58)	17 (77)	31 (67)	13 (57)	18 (78)	31 (67)
**Children and youths**									
No.	216	173	389	177	154	331	163	135	298
Female	103 (48)	79 (46)	182 (47)	87 (49)	71 (46)	158 (47)	80 (48)	67 (50)	147 (49)
Age, mean (SD), y	8.9 (2.9)	9.1 (3.0)	8.9 (2.9)	8.9 (2.8)	9.1 (3.0)	9.0 (2.8)	9.0 (2.7)	9.0 (2.9)	9.0 (2.8)
Fair or poor health as rated by parent	29 (13)	24 (14)	53 (14)	20 (11)	19 (12)	39 (12)	20 (12)	19 (13)	39 (12)
SDQ total problems score at baseline, mean (SD)	20.1 (3.8)	20.0 (4.0)	20.2 (3.9)	20.2 (3.9)	20.0 (4.1)	20.1 (4.0)	20.1 (3.9)	20.0 (4.0)	20.1 (3.9)
Saw mental health professionals in 6 mo prior to study, other than at a participating CMHC	36 (17)	31 (19)	67 (18)	32 (18)	30 (20%	62 (19)	31 (19)	24 (18)	55 (19)
**Accompanying adult**									
No.	216	173	389	177	154	331	163	135	298
Female	204 (94)	156 (90)	360 (92)	167 (94)	143 (93)	310 (94)	153 (94)	125 (93)	278 (94)
Age, mean (SD), y	37.0 (6.7)	37.9 (6.5)	37.4 (6.6)	36.9 (6.8)	37.8 (6.5)	37.3 (6.6)	36.9 (6.8)	37.8 (6.5)	37.6 (6.7)
Mother of child	199 (92)	147 (85)	346 (89)	163 (92)	134 (87)	297 (90)	150 (92)	119 (88)	269 (90)
GHQ total score at baseline, mean (SD)	29.1 (13.7)	29.1 (15.1)	29.1 (14.3)	29.1 (13.6)	28.8 (15.2)	29.0 (14.4)	28.8 (13.4)	28.7 (15.3)	28.7 (14.3)
First visit with GP	48 (22)	34 (19)	82 (21)	43 (24)	28 (19)	71 (22)	37 (23)	23 (17)	60 (20)
Family does not have enough money for expenses	91 (42)	86 (49)	177 (46)	77 (44)	74 (48)	151 (46)	65 (40)	63 (47)	128 (43)

Abbreviations: CMHC, community mental health center; GHQ, General Health Questionnaire; GP, general practitioner; SDQ, Strengths and Difficulties Questionnaire.

^a^ Randomization was balanced on these GP characteristics.

A total of 1170 eligible child-parent pairs were approached from May 2018 through February 2019, when the sample size for SDQ positive children was achieved; 619 of those eligible saw intervention GPs and 551 control GPs. Of these, 1111 provided consent and were screened (589 in the intervention group and 522 in the control group [95% for both groups]). One patient seeing an intervention GP could not be screened because of incomplete data; 4 parents seeing control GPs initially consented but declined screening; 1106 parents (588 seeing intervention GPs and 518 seeing control GPs) completed the SDQ screening. A total of 216 children (37%) seeing intervention GPs and 173 (33%) seeing control GPs screened positive (total, 389). Intervention GPs ultimately had from 1 to 20 screen-positive children and control GPs from 2 to 15 (Figure 1).

Child participants seeing intervention and control GPs were similar. The children’s mean (SD) age was 8.9 (2.9) years (range, 5-15 years), 182 (47%) were female patients, and their mean (SD) SDQ total problems score was 20.2 (3.9; range, 15-31) (Table 1). Approximately 18% (67 patients) had seen a mental health professional in the 6 months prior to screening. Parents seeing intervention and control GPs were also similar. Most parents were children’s mothers (346 [89%]). Parents’ mean (SD) age was 37.4 (6.6) years (range, 23-66 years), and their mean (SD) GHQ score was 29.1 (14.3; range, 1-74). Nearly half (177 [46%]) reported not always having enough money for expenses. Most had seen the participating GP at least once previously.

### Follow-up, Potential Confounders, and Primary Outcome

#### Follow-up

Of the 216 children seeing intervention GPs and 173 seeing control GPs, 177 intervention (82%) and 154 control (89%) patients were assessed at 3 months, and 163 (76%) and 135 (78%), respectively, were assessed at 6 months. Table 1 shows the number and characteristics of GPs, parents, and children at each of the 3- and 6-month points in addition to at baseline. No patients left the study because of adverse events. Loss to follow-up was not associated with GP characteristics (intervention group, practice size or financing, or gender) except that children seen by GPs in practice 10 or more years were more likely to be lost (eTable 5 in Supplement 2).

#### Potential Confounders

In bivariate analyses, older child age (≥11 vs <11 years) and greater GP length of time practicing at their site (≥10 vs <9 years) were associated with larger decreases in SDQ scores (eTable 1 in Supplement 2). GP gender (female vs male) was associated with relative increase in SDQ scores. Female GPs tended to be in practice a shorter length of time than male GPs (50% of female GPs in practice ≥10 years vs 83% for male GPs).

#### Primary Outcome

SDQ total problems scores decreased among children seen by both intervention and control GPs taken as a whole (Table 2; eTable 2 in Supplement 2). Adjusted for clustering within GP, the variables used for balanced allocation (practice size, practice ownership, and study wave), and the other variables associated with change in SDQ scores over time, there was not a significant time-treatment interaction at either the 3- or 6-month follow-up points (linear combination of coefficients for intervention, 0.57 [95% CI, –1.07 to 2.22] and –0.08 [95% CI, –1.76 to 1.56], respectively). Using the same adjustments, the intervention was not associated with significantly greater improvement in any of the SDQ subscale scores. eAppendix 2 in Supplement 2 demonstrates that similar results were obtained through alternative approaches to analysis (outcomes modeled as post-pre change in individual child SDQ scores, post-pre change in mean SDQ score per GP, interaction of mean SDQ score per GP with time, time modeled as a continuous variable).

**Table 2.** Mixed-Effects Multilevel Regression Results for SDQ Total Problems Score

Variable	Coefficient (95% CL)		
	Model 1 (group and time only)	Model 2 (group, time, balancing covariates)	Model 3 (group, time, balancing covariates, moderators)
Intervention vs control	–0.11 (–1.66 to 1.43)	–0.14 (–1.61 to 1.32)	–0.53 (–2.13 to 1.07)
Time point: 3 mo vs baseline	–3.50 (–4.34 to –2.66)	–3.50 (–4.34 to –2.66)	–3.52 (–4.35 to –2.68)
Time point: 6 mo vs baseline	–4.51 (–5.39 to –3.64)	–4.51 (–5.39 to –3.64)	–4.53 (–5.41 to –3.65)
Group × intervention interaction			
Intervention, time point 1	1.09 (–0.05 to 2.23)	1.09 (–0.05 to 2.24)	1.10 (–0.04 to 2.24)
Intervention, time point 2	0.46 (–0.73 to 1.64)	0.45 (–0.73 to 1.63)	0.45 (–0.74 to 1.63)
Public vs private practice	NA	–0.10 (–1.84 to 1.63)	–0.81 (–3.12 to 1.51)
Large vs small practice	NA	0.60 (–0.75 to 1.94)	0.90 (–0.63 to 2.43)
Study wave			
Wave 2 vs 1	NA	0.48 (–1.16 to 2.13)	0.27 (–1.54 to 2.08)
Wave 3 vs 1	NA	–1.19 (–3.08 to 0.69)	–0.83 (–2.93 to 1.28)
Parent GHQ positive at baseline	NA	NA	1.51 (0.67 to 2.34)
Child ≥11 y vs <11 y	NA	NA	0.21 (–0.70 to 1.12)
GP at site ≥10 y vs <10 y	NA	NA	–0.80 (–2.91 to 1.30)
GP female vs male	NA	NA	0.51 (–1.28 to 2.30)
Constant	20.14 (19.00 to 21.27)	19.94 (18.34 to 21.53)	19.51 (16.91 to 22.11)
Linear combination: intervention at baseline plus			
Intervention × 3 mo interaction	0.98 (–0.61 to 2.57)	0.95 (–0.56 to 2.46)	0.57 (–1.07 to 2.22)
Intervention × 6 mo interaction	0.34 (–1.28 to 1.97)	0.31 (–1.24 to 1.85)	0.08 (–1.76 to 1.56)

Abbreviations: CL, confidence limits; GHQ, Generalized Health Questionnaire; GP, general practitioner; NA, not applicable.

### Secondary Outcomes

#### Mental Health Services During the Study Period

Adjusted for clustering within GP, for seeing a mental health professional prior to baseline, and for the variables used in study allocation, children and youths seeing intervention GPs had about a 3-fold increased odds (odds ratio, 3.0; 95% CI, 1.1-7.7) of seeing a mental health professional during the course of the study compared with children seeing control GPs (Table 3). Table 3 also shows the secondary outcomes measured as a proportion of patients (or parents) with the outcome per GP. A risk difference (intervention GPs vs controls) with 95% confidence limits and a number needed to treat are presented for each secondary outcome. This method of analysis, which was not planned, found that all of the parent- and GP-reported secondary outcomes other than parent GHQ score had statistically significant 95% confidence limits.

**Table 3.** Secondary Outcomes: Crude Percentages and Adjusted Odds Ratios

Outcome	Participants, No./total No. (%)		Odds of outcome in intervention vs control group, odds ratio (95% CI)^a^	Difference in proportion of patients with outcome, intervention vs control GPs (95% CL)	No. needed to treat (1 / difference in proportion)
	Intervention	Control			
Parent reports of mental health services received during study period^b^					
Child saw mental health professional during study^c^	59/162 (36)	32/136 (24)	3.0 (1.1 to 7.7)	0.13 (0.10 to 0.17)	7.7
Parent saw mental health professional during study^c^	33/161 (21)	22/137 (16)	2.1 (0.8 to 5.5)	0.08 (0.05 to 0.10)	12.5
Parent reports of problems discussed with GP at baseline visit					
Any child psychosocial problem	149/214 (74)	105/168 (63)	2.0 (0.9 to 4.8)	0.12 (0.07 to 0.18)	8.3
Child behavior	149/215 (69)	102/168 (61)	1.8 (0.8 to 4.3)	0.10 (0.05 to 0.16)	10.0
Child’s emotions	149/215 (56)	95/168 (57)	2.2 (0.9 to 5.9)	0.14 (0.09 to 0.20)	7.1
Child’s problems at school	128/212 (61)	77/168 (46)	2.1 (1.1 to 4.2)	0.15 (0.10 to 0.20)	6.7
Child’s social interactions	115/215 (56)	72/168 (44)	1.8 (0.9 to 3.6)	0.12 (0.07 to 0.17)	8.3
Parent’s own psychosocial issues	141/215 (66)	82/167 (49)	2.1 (1.1 to 3.8)	0.19 (0.14 to 0.23)	5.3
GP reports of observations and actions related to child’s mental health at baseline visit					
Believes child has mental health problem requiring treatment	126/216 (59)	89/173 (51)	1.6 (0.8 to 3.0)	0.07 (0.03 to 0.12)	NA
Referred for mental health care	60/216 (28)	30/173 (17)	2.2 (0.9 to 5.5)	0.11 (0.07 to 0.15)	9.1
Counseled about mental health, if believes had mental health problem	94/216 (44)	55/173 (32)	1.8 (1.02 to 3.3)	0.12 (0.08 to 0.16)	8.3
Prescribed medicine for a mental health problem	8/216 (4)	12/173 (13)	0.70 (0.2 to 2.9)	–0.03 (–0.05 to –0.01)	29.6

Abbreviations: CL, confidence limit; GP, general practitioner; NA, not applicable.

^a^ Adjusted for clustering within GP and variables used to balance GP study status allocation.

^b^ Based on report at final (6-month) time point; denominators reflect patients followed to that time point.

^c^ Also adjusted for having had mental health visit prior to study.

#### Parent Reports of Interactions With GPs at the Index Visit

Parents seeing trained GPs were significantly more likely to say that their own psychosocial issues had been discussed at the index visit compared with parents seeing control GPs (OR, 2.1; 95% CI, 1.1-3.8). More parents assigned to the intervention group reported that GPs discussed children’s psychosocial problems at the study’s index visit, although of the 4 categories of child issues measured (behavior, emotions, school, and social interactions), only the increase in the odds of discussing school issues reached statistical significance (OR, 2.1; 95% CI, 1.1-4.2) (Table 3).

#### Change in Parental Mental Health

GHQ total scores decreased among parents in both groups (eTable 3 in Supplement 2). Adjusting for clustering within GP plus other variables associated with change in GHQ scores (eTable 3 in Supplement 2), there was not a significant effect of treatment allocation on parent GHQ at either the 3- or 6-month follow-up points (coefficients for intervention, –2.1 [95% CI, –4.9 to 0.7] and –1.6 [95% CI, –4 .5 to 1.3], respectively).

#### GP Detection of and Response to Mental Health Problems

There was little difference in the proportion of children and youths that GPs identified as having a treatable mental health problem (126 [59%] in the intervention group vs 89 [51%] in the control group) (Table 3). However, GPs in the intervention group were more likely to report that they had counseled the family about a child mental health problem (odds ratio, 1.8, 95% Cl, 1.02-3.3; adjusted for clustering within GP and the allocation variables). Compared with control GPs, more intervention GPs said they referred children with mental health problems and fewer said they had prescribed medication, but these differences were not statistically significant.

### Exploratory Subgroup Analysis

**Figure 2.**
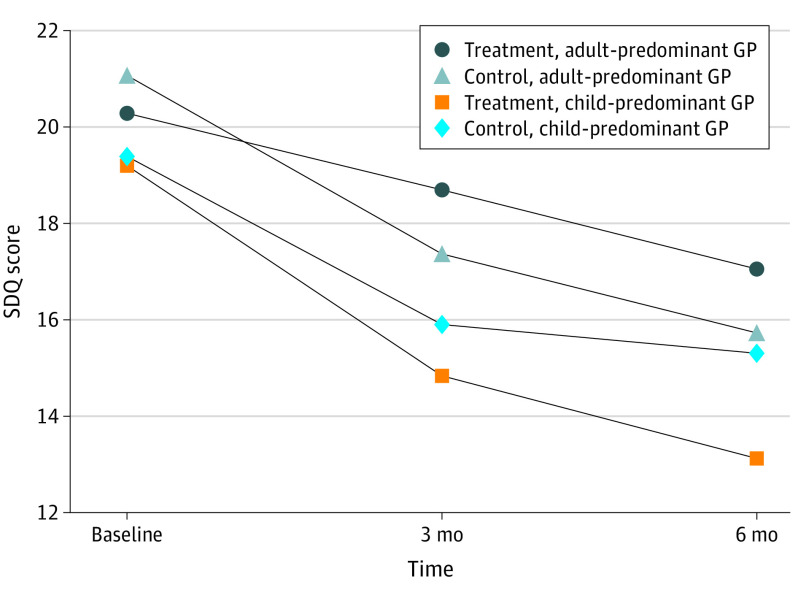
Adjusted Strengths and Difficulties (SDQ) Total Problems Score by Intervention Group, Time, and General Practitioner (GP) Practice Type

No significant treatment-subgroup interactions were found among subgroups defined by parental GHQ status at baseline, children’s age, or children’s gender. However, there was a statistically significant interaction of treatment with GP practice composition (child- vs adult-predominant) (Figure 2; eTable 4 in Supplement 2). From the multilevel model with a 3-way interaction term for treatment, time, and practice composition, the intervention-control difference in SDQ scores at 6 months was significantly larger among children seen by child-predominant GPs compared with children seen by adult-predominant GPs (–3.6 points; 95% CI, –6.7 to –0.46 points). As for the GPs overall, among child-predominant GPs, the intervention was associated with greater odds of the GP counseling (OR, 3.3; 95% CI, 1.0 to 10.3). Similar results were found from the alternative methods of analysis presented in Supplement 3.

To estimate an effect size within the subgroup of GPs in child-predominant practices, we computed baseline to 6-month SDQ change scores. Using the same variables for adjustment as noted previously, the mean decline was 6.9 (95% CI, 5.5-8.3) points for children seen by intervention GPs and 3.6 (95% CI, 2.5-4.7) points for children seen by control GPs, producing an effect size of *d* = 0.66 (95% CI, 0.30 to 1.01). Within the bounds of our sample size, we could not demonstrate differences between child- and adult-predominant GPs with regard to characteristics measured, nor could we demonstrate differences among children and parents.

## Discussion

We found that a brief training for generalists in collaborative care increased the odds that children and youths with positive SDQ scores would receive mental health care when they came for routine medical visits but did not result in greater improvement of child Strengths and Difficulties Questionnaire scores (the primary outcome measurement). GPs in both study groups were trained to recognize child and youth mental health problems and did so similarly. But intervention GPs were more likely to say that they had counseled about child mental health problems, and parents seeing intervention GPs were more likely to say that the GP had discussed parent and some child psychosocial problems.

We do not know why, despite this increased care, that training only seemed to improve child outcomes among GPs in child-predominant practices. Their counseling could have been more impactful if they were able to incorporate prior knowledge of children with what was taught in the training. The child-predominant GPs may also have had more positive attitudes toward caring for children and families and communicated this positivity to parents. Studies of GPs with special interests have found that their attitudes and confidence are, understandably, shaped by their prior experiences.^32^ Conversely, we found that the trained adult-predominant GPs’ patients improved the least. It may be that these GPs relative lack of experience with child and youth problems could have led to treatment attempts that were off-putting to parents and children.^33^ If replicated, this effect could call for caution when trying to promote child and youth collaborative care among clinicians with limited experience working with children and their families.

Among the GPs overall, female gender and longer time in practice had opposite influences on child outcomes. We suspect that social change and emigration from LMICs could result in cohorts of GPs with divergent training and practice styles.^34^ Neither of these GP characteristics, however, was related to response to training.

### Limitations and Strengths

This study has limitations. It was not powered to examine all the complexities of collaborative care.^35^ We do not know the specific content of visits, and adult-predominant GPs might have caught up to their child-predominant colleagues with additional training, practice, and coaching. Although we followed up families for only 6 months and further improvement might have been seen later on, reports of outpatient care for common child and youth mental health problems suggest that the most rapid phase of improvement takes places within 3 to 4 months.^16^

We caution, too, that our study took place within an existing collaborative and might not generalize to GPs overall or to other collaboratives. Brief trainings might not benefit generalists not already comfortable with adult mental health. Additionally, the results might not translate to clinicians who care only for children, since the GPs’ attention to parents might have played a role in children’s improvement. Conversely, had screening been a routine clinical practice within the collaborative, detection rates and child outcomes might have been better.^36^

Strengths include enrolling a relatively large number of clinicians of a type commonly providing primary care in countries across the income spectrum^37,38^ and demonstrating an impact on a heterogeneous population of children. We were also able to document improvements despite living conditions that worsened markedly during the study.^39^

## Conclusions

In this cluster randomized trial, GP training on managing common child mental health problems did not demonstrate greater improvement in child SDQ scores. However, the findings suggest that adding a child component to adult collaborative care may improve service access, but not all generalists may benefit equally from child training. As mental health training for generalists spreads, identifying clinician characteristics associated with patient outcomes and tailoring training to clinicians’ capabilities may help make the most efficient use of scarce training resources.

## Notes

Supplement 1.
Trial Protocol

Supplement 2. **eAppendix 1.** Data Analysis Plan and Sample Output **eTable 1.** Potential Confounders’ Relationship to Primary and Secondary Outcomes, Adjusted for Clustering With GP **eTable 2. **Crude SDQ Total Problems Scores by Time and Treatment Group, Adjusted Coefficients for Treatment Effect by Time **eTable 3. **Crude GHQ Total Scores by Time and Treatment Group, Adjusted Coefficients for Treatment Effect by Time **eTable 4. **Crude SDQ Total Problems Scores by Intervention Group, Time, and Child- or Adult-Predominant GP Practice, Adjusted Coefficient for Difference in Group-Time Interaction by Practice Type **eTable 5. **Baseline Factors Related to Children/Parents Being Lost to Follow-up at 6 Months **eAppendix 2.** Sensitivity Analyses: Alternative Approaches to Analysis

Supplement 3. Data Sharing Statement
