# Supplementary material for: Effect of General Practitioner Training in a Collaborative Child Mental Health Care Program on Children’s Mental Health Outcomes in a Low-Resource Setting: A Cluster Randomized Trial
Source: JAMA Psychiatry. 2022 Nov 30;80(1):22–30. doi: 10.1001/jamapsychiatry.2022.3989 (PMC9713683; doi:10.1001/jamapsychiatry.2022.3989)
Supplement: Supplement 1. — Trial Protocol [file jamapsychiatry-e223989-s001.pdf]

Iran/TUMS collaborative care trial protocol
Final version April, 2019

Overview: This application is for the second, trial component of an NIMH-funded study that will build and test (with a hybrid implementation-effectiveness design) a collaborative child mental health program in Iran. The project involves investigators in the JHU SPH, University of Washington, and partners at the Tehran University of Medical Sciences (TUMS).

The overall project has been reviewed and approved by the ethics committee of the Tehran University of Medical Sciences IRB (English translation attached). Some recently-modified design elements reflected in the protocol below were submitted to and accepted by the TUMS IRB – they do not provide an updated approval. The initial formative phase of the work was approved as JHU IRB00111549. This trial phase was registered with ClinicalTrials.gov as NCT03144739 on 5/8/17.

Most mental health problems begin in childhood and adolescence, but delays in receipt of treatment are measured in years to decades (Wang 2004, 2007). As a result, mental health problems cause a disproportionate burden of disability among children and youth compared to adults, and have a major impact on life course development (Kieling 2011). Primary care services can play an important role in efforts to prevent and intervene early in the course of child and adolescent mental health problems (WHO 2003). Primary care services are widely available and offer an opportunity to interact simultaneously with children and their parents, treat mental health in the context of medical and developmental concerns, and reduce the stigma associated with visiting identifiable mental health facilities. While research with adults has shown the feasibility of integrating mental health care into primary care settings, and that it reduces the burden of mental illnesses, there have been few studies among children and youth. Evidence remains lacking that integration is feasible in diverse settings and that it improves clinical outcomes.

One essential element of integration is "shifting" first-line mental health engagement and treatment tasks to primary care providers (Patel 2013). Task shifting requires mental health interventions that fit the context of primary care services and the nature of the problems seen (Aarons 2011). To date, most adult and child integration models have targeted single conditions at diagnostic levels and relied heavily on additional co-located personnel to provide treatment. However, to achieve goals of prevention and early intervention, integration models for children and youth must take into account how the epidemiology of mental health problems in children and youth differs from that of adults. The symptoms of emerging child and youth problems often suggest multiple possible disorders and can be co-morbid with developmental and parental disorders. By definition, emerging problems amenable to early intervention are likely to be "subthreshold" and not qualify for treatment in a specialty setting, especially when resources are scarce, even though early intervention holds the promise of preventing progression (Cross 2014).

The purpose of this project is to test the effectiveness of adding a child/youth mental health component tailored to fit the context of primary care into an existing collaborative care program supporting primary care management of adult mental health problems. The rationale for carrying out the project in Iran is two-fold: first, while integration of child mental health with primary care offers opportunities to fill service gaps even in high resource countries, it is particularly important in low and middle income countries where child mental health resources are in even shorter supply and where other community-based services (such as those in schools) are also uncommon; second, Iran is typical of many countries in that it has well-developed, family-

oriented primary care and adult mental health systems but very little child mental health capacity; and third, one of the investigators has created and directs an adult mental health-primary care integration program onto which a child program can be efficiently added, allowing coordination of adult and child services at the primary care level.

Specific objectives

The specific goal of this application is to conduct a hybrid effectiveness-implementation trial with general practitioners in Iran focusing on the following questions:

1. Whether delivery of the tailored interventions by primary care providers results in improved child and parent mental health outcomes; and exploring the mechanisms by which the interventions achieve those outcomes (which conditions are more likely to be identified and treated by GPs, which treatments have the greatest uptake by parents and youth).
2. Whether a coordinated program of training, ongoing coaching, and collaborative care results in uptake of the tailored intervention by primary care providers as evidenced by treatment provided in primary care and participation in collaborative care through consultation and referral.

Background

Worldwide, from 10 to 20% of children and youth develop mental health problems (Kieling 2011). Neuropsychiatric problems are the leading cause of disability among young people. Because they frequently go untreated for years or decades, they create enormous burdens of disability, especially in countries with predominantly young populations (WHO 2003). However, commonly-occurring problems such as anxiety and depression can potentially be prevented or ameliorated through intervention in childhood and adolescence (Cuijpers 2005, 2008). The reviews and expert panels conducted for the WHO's Mental Health Gap Action Plan (mhGAP) supported interventions, delivered in general medical care, to detect and manage developmental disabilities, behavior problems, and depression (Patel 2013).

While school and community-based programs are also important to comprehensively approach child mental health prevention and early intervention, primary care has an important role for detection and treatment (Patel 2013). Primary care may be critical for coordination and access to services (WHO 2012). It is widely available and allows simultaneous interaction with children and their parents, treats mental health in the context of medical and developmental concerns, and reduces the stigma associated with visiting identifiable mental health facilities. However, to date, nearly all adult and child models for integration of mental health with primary care have targeted single conditions at diagnostic levels and relied on additional co-located personnel. While this has demonstrated the feasibility of integration on a limited scale, it stops short of providing a readily disseminated model for broadly addressing child mental health problems, which are often subthreshold, highly co-morbid, and can involve simultaneous treatment of parents. Countries across the spectrum of income and resources need integration models that address the range, complexity, and local idioms of child mental health problems, and that can be practically and effectively delivered by primary care providers themselves (Kieling 2011; Ventevogel 2014).

Elaborations on Wagner's Chronic Care Model (CCM) (Wagner 1996) have been the basis of most programs to integrate mental health with adult and pediatric primary care. Several CCM interventions with positive outcomes have been reported for adult depression; one positive trial has been reported for adolescent depression and one for disruptive behaviors among younger children, both using co-located personnel (Asarnow 2005, Kolko 2014). The first step in adapting the CCM for child/youth mental health involves developing the core set of engagement and self-management interventions: universal and problem-specific interventions that are

evidence-informed, suit the patients and their problems in any given community, and are feasible in general medical settings (Wissow 2008). First, the "common factors" literature from psychotherapy emphasizes how patient-provider interactions influence outcomes across diverse therapies and inform the CCM's engagement and coaching strategies (Wissow 2011). Studies in LMICs also find that patient-provider interactions (Labhardt 2009) impact outcomes in ways similar to what is observed in higher resource countries. Second, the "common elements" of evidence-based child mental health treatments suggest interventions (Chorpita 2005) that can be matched to child and parent concerns and delivered prior to completion of a formal diagnostic process (McGarry 2008). Third, studies of "single session" psychotherapy (and other brief intervention models among adults) demonstrate the effectiveness of providing treatment in brief pulses across extended periods, similar to patterns of medical primary care (Perkins 2008). Fourth, "stepped care" models for treating adult depression suggest that generalists can provide first-contact mental health treatment if they reliably follow patients to ascertain need for further diagnosis or intervention (Katon 2010). Finally, in both mental health and medical services, effective care involves leveraging community resources to support functioning (Davis 2010). In a study in the US, we found that training community providers in "common factors" skills impacts children's outcomes (Wissow 2008, 2011); we have preliminary evidence that this approach to integration can be successfully used for care of adults with HIV in Ethiopia (Wissow 2014; Jerene [in press]).

<u>Tehran University Collaborative Care Program (TUCCP):</u> In 1988, the Iranian Ministry of Health and Medical Education (MOH) set a goal of involving all rural and urban primary care centers in the detection and treatment of common and severe mental disorders. The program succeeded for adult services in rural areas (Khadivi 2012), but its reliance on community health workers (CHWs) failed in urban areas where populations were more mobile and less close-knit (Yasami 2001). In response, the MOH financed a pilot urban mental health collaborative care program, TUCCP, replacing CHWs with GPs as the first line of services and point of access to specialists (Sharifi 2011, 2014, 2015; Moinfar 2016). Led by the Iranian co-investigator of this application, TUCCP spans public and private primary care sites linking over 60 urban GPs with community mental health centers (CMHCs) for consultation, referral, and ongoing support of GP skills. For adults, the model promotes task-shifting care for common mental disorders to the GPs, and stepped care via referral for individuals with more serious mental disorders. However, for children and youth, the current model calls for GPs to function only as gateways, referring all levels of problems to a CMHC.

TUCCP is a sophisticated program that fully operationalizes the CCM and includes features advocated for US medical homes and accountable care organizations (Katon 2011). Its main components are: 1) an initial 3-day training followed by quarterly boosters and ongoing interaction between CMHCs and GPs, 2) informal mental health consultation to GPs about specific patients, including facilitating referrals to CMHCs (who may take over care or return the patient to the GP), 3) a case manager role for the GPs' receptionists, who telephone enrolled patients to check on their status, re-enforce interventions, and remind them of appointments, 4) a health information system for ongoing case tracking and quality assurance, and 5) payment of top-up fees to the GPs (like care management fees for US certified medical homes), contingent on documented follow-up and adequate treatment. When a GP identifies a patient with a mental health problem, he/she or the receptionist enters demographic and diagnostic information and the treatment plan into the information system. A psychologist and psychiatrist from the CMHC regularly call GP offices to monitor follow-up and give feedback on treatment plans and every 3 months visit offices to audit a sample of charts and discuss management. TUCCP also regularly calls a 5% sample of enrolled patients to ask them about satisfaction with the services they have received from GPs and their receptionists.

TUCCP and child services: Presently, TUCCP focuses on collaborative care for adults. Physicians participating in the TUCCP are advised to refer identified children/youth with mental health problems to CMHCs for further care. In contrast to the volume of adults, only 192 child/youth patients were enrolled in TUCCP's first 30 months, substantially fewer than expected, and only 67% completed referral to the CMHC. TUCCP leaders attributed this under-utilization in large part to the mandatory referral of child cases to CMHCs, which constituted a disincentive for both parents and GPs. In preparation for this project, TUCCP surveyed a sample (n=27) of participating GPs to explore their interest in additional child mental health training; *78% said they would likely participate*. GPs believed that a majority of parents would welcome alternatives to referral to CMHCs. They said that parents declined CMHC referrals because of distance, stigma, and concern that specialists would propose medication rather than psychotherapy. Over 75% of patients GPs referred had conditions (ADHD and anxiety) that could be treated in primary care.

Study procedures

Design, sequence and timing of procedures: In this phase of the work we will conduct a hybrid effectiveness-implementation trial (Curran 2012) to develop preliminary evidence of the feasibility of training GPs to address child/youth mental health problems and mechanisms leading to training uptake and outcomes. The trial is a staggered-start parallel design in which the recruited GPs will be randomly assigned to one of three groups, and then within these groups assigned to either intervention or control status. Intervention GPs will receive the child mental health training that is the subject of the study; control GPs will receive a child mental health "booster" (focusing on identification and referral) as an attention control. At the end of each group's study period the control GPs will have the opportunity to take the training received by the intervention arm. In the intervention arm, after training, if GPs believe a child/youth has a mental health problem, the GP will have the option to offer a) treatment him or herself, b) treatment in the GP office but with a referral for a one-time evaluation or c) referral to the CMHC as is currently the standard. For all cases in which the GP believes that the child/youth has a mental health problem, whether in the intervention or control group, office receptionists will register the child with the TUCCP, stay in contact with the family, re-enforce the GP's advice (whether that involves an intervention directly prescribed by the GP or involves a referral to the CMHC), and check on follow-through. All of these steps are routine parts of TUCCP care and are documented in the TUCCPs routine on-line data system.

To examine the impact of the intervention, in the first 1-2 weeks following training, cohorts of children 5-15 years old will be recruited and screened using the parent report version of the Strengths and Difficulties Questionnaire (SDQ) that corresponds to the age of their child. The SDQ has been validated in Iran and yields measures of symptoms, functioning, and family burden plus overall scores and domain scores for hyperactivity and emotional, peer relationship, and conduct problems. In studies in other countries, the SDQ has been found to have similar or better sensitivity and specificity as the much longer CBCL (Goodman 1999). Screening results will not be shared with the GP, and summary scores will not be discussed with parents. Parents and children/youth will be free to discuss the screen with the GP or anyone else, and RAs administering the screens will encourage parents to discuss any concerns they have with their GP. Those children/youth who screen positive will be followed by telephone with re-assessment at 3 and 6 months after screening (regardless of whether or not the GP has considered them to have a mental health problem and entered them in the TUCCP registry).

The following figure is the currently planned timeline for the study, assuming recruiting of GPs in February-March, 2018 and beginning the trial in March-April, 2018.

```
Study year        2016  7     |2017  8     |2018  9     |2019
month             JASONDJFMAMJ|JASONDJFMAMJ|JASONDJFMAMJ|JASO
Formative work    XXXXXXXXXXX
Pilot training               X|XX
Recruit GPs                           X
GP group 1                             TRFF|FFFFW
GP group 1                             CRFF|FFFF
GP group 2                               TR|FFFFFFW
GP group 2                               CR|FFFFFF
GP group 3                                 |TRFFFFFFW
BP group 3                                 |CRFFFFFF
Crossover trainings                        |    T T T

GP=general practitioners
R=Recruit patients: assume that in a month with R that some will be followed for part
of that month but still need six full months after
C=Control training
T=Intervention training
F=FINAL F/U (blue)[note that exact end date will depend on 6-month date of last-
enrolled patient
Three month follow-up – doesn't have a letter – yellow
W= wrap up qualitative interviews with parents and GPs
```

Population and recruiting:

1. Children/youth and their parents: The main participants in the trial are children/youth 5-15 years of age (male and female) and one of their parents or guardians (whoever accompanies the child/youth to their GP visit). These children will generally be coming to the GP for a non-emergent medical, emotional, or behavioral concern. Some children may have a chronic medical or mental health condition, but we will not recruit any child/youth who reports being in pain or who appears to be acutely ill. Children/youth who are actively being treated at the CMHC at the time of recruiting will also not be eligible. We plan to recruit approximately 1500 children/youth and 1500 parents (screening step) and follow about 350 of each (children who are SDQ+ and their parent) by telephone at 3 months and 6 months after the index visit.

The required patient sample size was estimated in four stages. We first used the "change" option in the "sampsi" routine in Stata Release 12 (StataCorp, College Station, TX 2011) to obtain a sample size disregarding clustering among general practitioners. The "change" option computes a t test on the difference in change scores (outcome minus baseline) between the intervention and control groups. The variance is adjusted for correlations between the outcome measures and between the outcome and baseline measures. We estimated that the difference in change scores at the final assessment would be about 2.5 points on the SDQ total difficulties scale, based on data from a prior trial conducted in primary care in the US (Wissow 2011) and on data from the UK child and adolescent mental health care system (Wolpert 2015). An estimated standard deviation of 7.2 was also drawn from these sources (thus an estimated effect size of .34). Correlations between baseline and outcome measures and between outcome measures were estimated at .37 and .55, respectively, also based on prior data from the US (Wissow 2011). With alpha=0.05 and power of 0.8 these parameters yielded a sample size of 135 per study arm (270 total).

In cluster-randomized studies, sample size is dependent on the extent to which outcomes are correlated within clusters. A review of adult primary care studies from high-income countries found ICC's for depression outcomes ranging from .016 to .035; the range for the SF-36 mental health measure was .009 to .033 with an overall median of 0.01 (Adams 2004). Given that we plan to enroll 45 general practitioners we would have clusters of 6 patients per general practitioner yielding a design effect of 1+(6-1)*.01=1.05, or a sample size at outcome of about

284. Assuming a worst-case of 20% loss of patients to follow-up would require enrolling 355 patients.

There are varying estimates of the prevalence of high/abnormal SDQ scores (=>17). We found 24% of those screened to be in this range in a study of children 6-16 in primary care in the US (Wissow 2008); Alavi and colleagues (2010) found a prevalence of 26% using this scoring of the SDQ in a population-based sample of children 6-11 in Tehran. Using an estimate of 24%, we then need to screen about 1479 children/youth.

For screening, we will recruit consecutive patients as they come for clinic visits. Families will be approached by a research assistant when they arrive for visits and asked if they are interested. Families will not be approached if the child appears to be acutely ill or in pain. If the family is interested, they will be taken to a nearby private space to discuss and consider consent/assent. We anticipate that most families will complete baseline data collection by telephone to avoid delaying their visit and to assure privacy. If time and privacy permit, consenting families will have the option to complete data collection in person before or at the end of the visit. To facilitate telephone administration, families will receive blank copies of the main questionnaires to use, if they desire, as references when later answering over the phone. Families who consent will receive a small token (value about $2).

<u>2.GPs</u>: In this cluster-randomized clinical trial, the unit of randomization is the practice (which in most cases is the same as the GP, since most GPs are in solo practice). We will recruit from among GPs who are already taking part in the collaborative care program. These GPs are already tasked with detecting child and youth mental health problems but at present they are instructed to refer all such children to the CMHC. GPs will be randomized in a way that balances, to the extent possible, characteristics of their practice (volume, proportion of patients who are children/youth, prior participation in the collaborative care program, public/private practice). We will try to recruit all 60 GPs currently in the program, but have powered the study for 45.

GPs will be approached by staff from the CMHC with whom they already interact regularly. If they are interested in the study, one of the Iranian investigators will contact them, provide more information and obtain written consent. GPs will receive a payment of about $200 to cover the time that will be required for training in child/youth mental health skills. In addition, as in the current Tehran University Collaborative Care Program (TUCCP), they will receive a care coordination payment for patients identified as having a mental health problem who they successfully follow for at least three months (presently this applies only to adults – for participants in this trial it will apply children as well). Successful follow-up is defined as a) monthly visits with the GP for the first three months after treatment initiation or substantial change in treatment, and then b) follow-up visits at no more than 3-month intervals for patients who are stable. Patients for whom the GP is not certain of the diagnosis, or a) for whom there is concern for self-harm, harm to others, psychosis, mania, or a clinically important drop in function, or b) who cannot be stabilized after three months or may be having severe side effects to medications, are referred to the CMHC.

Data collection
Baseline data collection will take place at the time of enrollment or by telephone after the GP visit. Cognitive interviews and pilot testing will help assure that respondent burden is acceptable and achievable in a reasonably brief telephone call; should the plan proposed in this application require changes we will submit amendments to the protocol. The investigators have experience using the SDQ by telephone in Iran, and the GHQ (see below) by telephone in the US. The

research assistant administering the instruments will enter the responses into a secure electronic data base that will a) determine if the family is to be entered into the follow-up phase of the study (ie, child “positive” at the “possible” or “probable” level on the SDQ) or if responses to the parent self-harm question on the GHQ triggers notification of the GP. There will also be a brief exit survey for the parent and for older children/youth after the index visit. This will be administered by the research assistant in person at the GP office; however, as above, if lack of privacy or time do not permit, the survey can be administered by telephone.

The GHQ (General Health Questionnaire) is a brief instrument (28 items) used to rate adult emotional status. Parent mood is an independent predictor of treatment adherence and outcomes for children’s mental health services, and a separate target of TUCCP care. The GHQ can be used categorically with good sensitivity and specificity for psychiatric disorder, and as a continuous measure of distress. Its questions are framed in terms of recent changes in mood, which make it suitable for use in longitudinal studies, and it has been used in several studies in Iran (Emmami 2007).

Parents will be asked to completed the EuroQol EQ-5D-5L, a brief (6 item) instrument that measures five areas of function (mobility, self-care, usual activities, pain/discomfort, anxiety/depression) at five levels. It will offer us the opportunity to understand the impact of parental disability which may or may not include mental health concerns disclosed on the GHQ. The EQ-5D-5L can be administered by telephone and is sensitive to a wide range of chronic conditions including mood and chronic pain problems in general populations (Agborsangaya 2014). Unlike many instruments, the EQ-5D-5L is scored by comparing the pattern of responses across the five areas to a population-based data ranking the relative value respondents place on each pattern of disability. A Farsi version of the EQ-5D-5L has been developed and a value set for Iran has been developed to guide its interpretation (Kiadaliri 2015).

<u>Parent satisfaction with care</u> will be assessed by asking parents if the visit involved discussion of a mental health problem, and, if so, if the GP suggested a plan for treatment (Brown 2008). Subsequently we will ask about satisfaction using questions initially developed by Zastowny and colleagues (1983) based on Hulka’s (1970) conceptualization of satisfaction with providers’ interpersonal skills and technical competency. Two questions ask about overall satisfaction with the visit, three reflect satisfaction with informativeness (was the doctor's information helpful, did the doctor clearly explain what you should do, answer all your questions) and four reflect partnership (did the doctor encourage you to talk about your worries, ask for your opinion about treatment, spend enough time with you, treat you with respect). In past work (Wissow 1998) we found that while the overall satisfaction and informativeness items are skewed to higher ratings (a ceiling effect), the partnership items are sensitive to differences in provider interaction style, which is targeted by one of the components of the intervention training.

Table: Instruments completed by patients/family

Instrument name	Respondent	Timing/method of administration*
Strengths and Difficulties Questionnaire (33 items)	Parent about child	Baseline and 3 and 6 months later if SDQ+
General Health Questionnaire (28 items)	Parent about self	Baseline and 3 and 6 months later if SDQ+
Patient and family demographics (22 items)	Parent about self and child	Baseline
Health status EQ-5D-5L (6	Parent about self	Baseline and 6 months later if

items)		SDQ+
Satisfaction with GP interpersonal and technical skills [exit interview] (15 items)	Parent about self	Baseline
Use of community services (6 items)	Parent about self and child	Baseline and 6 months later if SDQ+

* note that all completed by telephone except some baseline instruments may be completed in-person at the time of the index GP visit

Treatment and control interventions.
All GPs will follow guidelines set by the collaborative care program. Control GPs, as is present procedure, if they identify a child or youth under 16 with a mental health problem, will be asked to enter that child/youth into the TUCCP data system and refer the child to the community mental health center. Parents are free to accept or decline the referral and the GP is free to provide counseling or prescribe a medication (other than stimulants, which can only be prescribed by a psychiatrist). Control GPs will receive a one-day child/youth mental health refresher course that will focus on identification of child and youth emotional and behavioral problems. The material will expand on past TUCCP trainings and cover only recognition and discussion with families about treatment options available through the CMHCs and will include role plays and interviews with standardized patients.

Intervention GPs will also enter cases they identify into the TUCCP database and have the option to refer immediately (especially if that is the family's preference). However, they will also formally have the option to provide treatment themselves, with or without informal consultation with the CMHC. Cases entered into the TUCCP database and treated by the GP will be monitored by CMHC staff via the database and periodic site visits for chart review (as is currently the practice for adults treated by GPs). At any point in the treatment the CMHC staff may suggest that the child/youth be referred to the CMHC. The TUCCP staff also monitors the database for children/youth referred to the CMHC but who have not completed a first visit. In those cases the receptionist is charged with encouraging the patient to attend the CMHC or to return to the GP for treatment. Intervention GPs will receive a two and a half-day training built around role plays and interviews with standardized patients. Training content is derived from past work, the WHO's mental health gap (mhGAP) program, and from information gained in the formative phase of the project. The intervention training is designed to help GPs identify child and youth emotional and behavioral problems, engage families in care, provide first-line interventions, and refer to the community mental health center based on family need or desire. The training takes a trans-diagnostic approach to mental health care, based on universally-applicable communication skills; broadly applicable techniques including problem solving, emotion regulation, and help with parent-child interaction; and use of problem-specific brief treatments drawn from mhGAP. Work in the formative phase led to the addition of substance problems and special attention to the needs of immigrant families to the previously planned material addressing children's mood and behavior.

As noted above, we will not share screening results with the GPs, though we will tell parents that they are free to talk with their GP about the screening and their answers. We have made this decision based on the pragmatic nature of this trial. Initiation of universal or even consistent indicated screening is technically challenging and, were we to be initiating it as part of the study, would not likely be continued. That would be a threat to the generalizability of our results. Future studies may be able to address the costs and benefits of routine screening being part of the support offered by participation in TUCCP.

Child/parent follow-up data collection. An abbreviated data set (the key mental health outcome measures – SDQ and GHQ) will be collected by telephone three months after the initial visit for both the control and intervention children/parents; a longer final data collection will occur at six months after the initial visit. We will only follow those families whose child screened positive on the SDQ at the index visit, or for whom the SDQ was negative but the GP entered the child in the TUCCP database. It will not be possible to guarantee that research assistants making follow-up calls will be blind to the child's control-intervention status because assistants who recruit families will, when possible, also make follow-up calls so that a known person is maintaining contact. However, the assistants will have no other information about the family other than what is reported on the study instruments. In particular, they will have no knowledge of any data about the child from the TUCCP database, and they will administer the SDQ and GHQ prior to instruments (at the baseline and final visit) that might reveal information about use of any mental health services. We believe that the families' familiarity with the research assistant, coming from an in-person contact at the time of consenting, will promote follow-up. We believe that this advantage balances out concern about biased outcome assessment.

The literacy rate varies among the families to be recruited, and we anticipate that some parents will require that the consent forms (and instruments if done in person) will be read to them. This will take place in private, and we will have a sufficient number of female research assistants so that there are no barriers to participation because of staff gender.

We will collect, and maintain separate from study data, basic contact information for child/youth participants and their parent/guardian. Cell phone penetration is nearly 100% in the population to be studied, but in the event that contact is not possible, research assistants will attempt contact by mail and/or by leaving a message at the GPs office and the community mental health center (CMHC) should the child have a follow-up visit. Contact information will be destroyed once all follow-up data have been entered into the study database and checked for quality and completion.

From the TUCCP data system, we will be able to determine the number of mental health cases identified pre- and post GP training, the number of follow-up visits for each identified patient and their site (GP office or CMHC), and the number of referrals (and referral completions) to the CMHC.

Child/parent follow-up interview: A small sample (16-24) of those child/parent pairs being followed in the intervention groups will be approached for qualitative interviews at three and six months into their observation period (this possibility will be included in their initial consent). The interviews will be scheduled at a time and place of the family's convenience; with parents' consent, youth will be given the opportunity to be interviewed alone. The interviews will focus on the experience of receiving care for emotional or behavioral problems from the GP. However, the interview will not imply that the child/youth has such a problem. It will simply refer to the issues the parent/youth have reported on the SDQ, which have been the subject of the study follow-up. We will use purposeful sampling to select families for follow-up interviews, with the sampling frame to be determined partly by child age, gender, and predominant symptoms on the baseline SDQ. We may sample according to other characteristics that emerge as important during the trial (for example, if there are many families that decline GP treatment). These interviews will be conducted by a two-person team, one of whom is assigned to take notes. They will not be recorded.

Summary of clinical data collection	
**Time point**	**Instruments**
Index visit at time of	Parent and child demographics, prior service use, GHQ, SDQ, reason for visit, EQ-

recruiting	5D-5L
Immediately after index visit	Parent exit questionnaire: Discussion of any mental health problem, follow-up plans, satisfaction with visit<br>Provider exit questionnaire: Suspected mental health problem (y/n), treatment plan (including use of collaborative service)
Three months after index visit	GHQ, SDQ
Six months after index visit	GHQ, SDQ EQ-5D-5L, other service use (includes TUCCP data about referrals and follow-up visits to GP or CMHC)

GP training

Training content is derived from past work, the WHO's mental health gap (mhGAP) program, and from information gained in the formative phase of this project. Interviews and focus groups with GPs, parents, youth, and school officials found several areas that required emphasis in addition to the material that we had planned to present. These included additional emphasis on substance problems, special attention to the needs of immigrant families, and areas in which the GPs (who already have training in adult mental health issues) were particularly uncomfortable (for example, child development, talking with children and parents about child mental health problems, and child abuse).

Training format was informed by a pilot training in another city (Tabriz) attended by 18 GPs. The training started and ended with GPs taking part in brief mock visits with standardized patients. These visits were well-received by the GPs (especially for making the training engaging and interactive) and re-enforced the need for training in how to communicate with families about child/youth mental health issues. Though GPs were most interested in topics unique to children (ADHD, development), they still demonstrated a need for understanding and being able to talk about the particular child/youth manifestations of issues with which they were already familiar from adult training (depression, anxiety).

Training is planned for a two and a half day in-person session followed by an in-person booster session a short while later. GPs will have continuous support from mental health center staff who will provide them with feedback about registered cases and who are available for informal telephone consultation. A "pocket guide" to key material in the training will also be developed for use either on paper or on line. GPs will receive the equivalent of about $200 as compensation for their time. As noted above, they will also receive an incentive payment from the collaborative care program (a standard part of the program, not part of the research) for successfully managed child cases (defined by the TUCCP).

Content areas for child/youth mental health training

**Topic**	**Brief interventions**
Patient-provider interaction	Engaging patients in conversation, attentive listening, eliciting an agenda, empathy, giving advice, decision support, interacting with child and parent ("Common factors")
Children with problematic behavior	Assessing possible causes (anxiety, depression, trauma, developmental delay or school problems, attention problems), general parenting advice, help with schoolwork, psychoeducation for families about children with delays; linking to community resources for children with developmental delays; appropriate medication use.
Depression (child and parental)	Psychoeducation, behavioral activation, exploration of contributing stresses, exploration of suicidal ideation, safety for suicidal patients, medications for older adolescents and parents.
Anxiety	Psychoeducation, active coping (role models, supported exposure, relaxation), support and response to maladaptive cognitions following trauma, exploration of contributing stresses, indications for medication.
Substance use	Recognition of harmful use of tobacco, alcohol, and inhalants. Brief counseling regarding cessation.

GPs assigned to the control arm of the study will receive a one-day child mental health update that will include role plays and practice recognizing child mental health problems. This will be a "booster" since similar trainings have been offered to GPs in the TUCCP in the past. As is

currently TUCCP policy, GPs will be instructed to refer all detected cases to the CMHC. At the end of the trial, all control GPs will be offered the intervention training.

<u>GP data collection.</u> The GPs will complete brief demographic and attitude surveys at the outset of the study and again at its conclusion. Both control and intervention GPs will complete very brief post-visit forms for enrolled patients indicating their assessment of whether a child has a mental health problem and, if so, their working diagnosis and treatment plan. They will also interact with two standardized patients portraying child mental health problems – one at the end of their training and once during the patient follow-up period.

Assessments completed by GPs

Instrument/assessment name	Respondent	Timing/method of administration
Demographic data	GP	Baseline recruiting of GP
Confidence in treating common child mental health problems	GP	Baseline recruiting of GP and end of intervention period
Confidence in treating common adult mental health problems	GP	Baseline recruiting of GP and end of intervention period
Burden subscale, Physicians' Belief Scale	GP	Baseline and end of intervention
GP visit exit questionnaire	GP	At end of index visits for consenting parent/child pairs
Standardized patient visits	GP	At end of initial training workshop and then once about midpoint of follow-up period

<u>GP follow-up interview:</u> A small sample (10-15) of participating GPs will be approached for follow-up qualitative interviews either in person or as a focus group (this possibility will be included in their initial consent). The interviews will be scheduled at a time and place of the GP's convenience. The interviews will focus on the experience of providing care for child/youth emotional or behavioral problems and collaborating with the CMHC. A major goal of the interview will be to understand how the experience of training and working with the CMHC was able to build GP confidence in treating these problems and willingness to do so. These interviews will be recorded and transcribed for analysis.

Inclusion/exclusion criteria:

1. Children and youth: age 5-15 years of age (male and female) and one of their parents or guardians (whoever accompanies the child//youth to their GP visit). These children will generally be coming to the GP for a non-emergent medical, emotional, or behavioral concern. Some children may have a chronic condition, but we will not recruit any child/youth who reports being in pain or who appears to be acutely ill. Children actively treated in the CMHC at the time of recruiting will also not be eligible.

2. GPs: Must be a current participant in the TUCCP collaborative care program and see children and youth as part of their practice. GPs who function solely as specialists will be excluded (some may have had a more general practice at the time they joined TUCCP).

Risks

Potential risks are listed below, with methods to address the risk detailed in the next section.

<u>1. Breach of privacy for children/youth and parents:</u> The information collected in the course of the study does not go beyond what could be collected in the course of routine general medical care that follows international guidelines for inquiry about psychosocial as well as somatic well-being. However, unwanted disclosure to third parties could be socially damaging or traumatic. Other than the loss of study data, the main threats to privacy are unwanted disclosure during attempts to follow-up on SDQ+ children/youth and unwanted disclosure of youth data to parents and vice-versa. We try to minimize these risks by training of staff regarding confidentiality and

security of data. In addition, no data collection other than the small number of follow-up interviews will involve information collected directly from children/youth (and even then they will not be the primary informants in the interviews), so instances in which youth may say something to a research staff member in confidence will be very limited. It is possible that some youth participants could disclose information to their GP that they might not want disclosed to their parents. In Iran, it is more common than in the US for teens or even adults to come for visits with a family member who remains present in the examination room during treatment. However, as in the US, GPs can choose to ask family members to step out to afford the patient privacy. In the other direction, it is possible that responding to the GHQ could prompt parents to want to discuss issues with the GP that they would also prefer to discuss in confidence.

The SDQ (for parents about their children) does not ask about self-harm or victimization, and it includes questions in which the parent is able to report positive attributes as well as problems. The GHQ (for adults only) does have one question about thoughts of self-harm but no questions about exposure to violence. No child/youth or adult instruments include questions about substance use, sexual orientation or any behavior that might be considered illegal or especially stigmatized in this community compared to any other. All three of the standardized instruments (SDQ, GHQ, EQ-5D-5L) have been previously used and found acceptable in studies in Iran. Though we will not ask about these things, when interviewed alone by their GP or at the CMHC youth may make statements raising concerns for maltreatment, self-harm, or a need for medical services about which they do not wish their parents to be aware. Except if it is discovered in the course of hospital treatment, Iranian law does not mandate child abuse reporting. Assent forms for youth will include a reminder that they may always ask to speak in confidence with their GP or another provider, though the decision to keep that confidence will rest with those providers.

<u>2. Privacy for GPs:</u> GP performance with standardized patients could subject their skills and knowledge to a level of evaluation that they would not ordinarily experience in the course of their regular practice (although these assessments are a regular part of TUCCP and GPs will have experienced them previously). All GP data will be treated confidentially.

<u>3. Coercion to consent and involuntary participation for children/youth and parents:</u>
Among the participants to be recruited, the children/youth by definition fall into the category of a potentially vulnerable population. However, parents may also be vulnerable for reasons of poverty, low literacy, or mental health problems of their own. We will make sure that the parents and children clearly understand that whether or not they choose to participate in the study will have no impact on the care they receive from the GP. We accomplish this by including clear language in the consent and assent and by employing research staff that are not employees of the practice.

In addition, this study uses a form of cluster-randomized design in which patients, who may have a pre-existing relationship with their GP that they do not want to disrupt, can knowingly or unknowingly find themselves being offered a different treatment (in this case, the doctor's interest in treatment their mental health problem him or herself rather than making a referral) (McRae 2011). Cluster-randomized trials in which providers or sites are the unit of randomization have been criticized because they do not allow patients a choice of treatment; consent serves only as notification that they may be receiving a different treatment and allows them to agree to participate in an evaluation. As we describe below, trained GPs in this study will not be obliged to treat patients themselves (they can still make referrals to the CMHC) and patients will be able to access the CMHC or other mental health services regardless of the GPs decision.

4. Coercion to consent and involuntary participation by GPs: GPs might feel obligated to participate in the new child mental health training in order to maintain their affiliation with TUCCP. As noted below, we will make it clear that participation is voluntary.

5. Risk of inferior treatment of children/youth and parents: Currently in the TUCCP, children that the GP believes may have a mental health problem are, by protocol, referred to a CMHC for treatment. This treatment could be superior to what a GP could offer. However, we believe that there is currently considerable under-identification of children with potentially treatable problems. In addition, as many as a third of children currently referred to CMHC's by TUCCP GPs do not complete the referral. Still, there remains the risk, following GP training, that GPs will attempt to treat children who should be referred, or will not realize that a child's condition has not responded to treatment in primary care and now requires referral. We minimize this risk by specifically discussing during the GP training scenarios in which the child needs to be referred to the CMHC. In addition, the TUCCP monitors treatment and follow-up as part of its routine processes and prompts GPs' to make additional follow-up attempts, modify treatment when needed, or refer to the CMHC.

6. Risk of additional work for GPs: GPs may face additional clinical work if their practices detect and manage more child or parent mental health problems. As noted before, there is reason to believe that child mental health problems are under-recognized in TUCCP practices; those that are recognized presently are referred. Off-setting additional work is the possibility that GPs could receive additional top-up payments for managing child mental problems, and that repeat visits for masked mental health problems could be reduced.

Mechanisms for reducing risk:

Oversight

1. Direct supervision for this phase of the study will be by the Iranian PI, Dr. Sharifi, a psychiatrist at TUMS. Site level research personnel will be thoroughly trained in good clinical practice, human subjects procedures, and the importance of being alert for and documenting adverse events, protocol deviations, and participant concerns. All of these will be immediately communicated to Dr. Sharifi and the US PI.

The US PI will be in regular contact with the Iranian investigators and staff. Presently the US and Iranian teams have joint calls every 1-2 weeks and frequent email exchanges during the week. Once data collection is underway there will be one call a week for monitoring processes and one every two weeks to discuss data quality. The US PI and co-investigator have made one trip to Tehran for planning this phase of the study and plan subsequent trips to a) check on the process of data collection during the baseline phase, and b) observe the GP training. These trips are always subject to changes in international relations; in 2017 the Iranian and US PIs had to meet in Dubai.

2. Recruitment and informed consent: As noted above, children/youth will be recruited when they come for GP visits with a parent or legal guardian. Parents/guardians will be asked for written consent and children/youth for assent or co-consent as age-appropriate. Although the age of majority in Iran is 18, Tehran University ethical guidelines call for co-consent for children/youth ages 8 and older. If there is a concern about literacy, research assistants, will be able to go over the consent verbally. The consent process will take place in a private area in the clinic where the patient is being seen. Details about the consent process as it relates to specific risks are discussed below.

GPs will be recruited by announcing, in the course of existing TUCCP activities, the availability of new child mental health training. Opportunities for discussion about the training itself and its

evaluation will occur at both group and individual meetings. GPs will be individually and confidentially asked to provide consent. GPs choosing not to participate in the study will be able to continue in the TUCCP as at present but none of their patients will be recruited.

<u>3. Overall data security:</u> Precautions against loss of or breach of collected study data will include making sure that no study data forms or files ever contain identifying information (except those kept temporarily for follow-up tracking) and that linkage files are protected and destroyed as soon as possible. If linkage files ever need to be transported they will move separately from data files. Identifiable data will never be stored on portable devices unless this is for the purpose of locking those devices in secure physical storage. Appropriate firewalls, encryption, and password protection will be used for network-connected devices used to store or manipulate study data.

Individually identifiable data will initially be accessible to research assistants and the study's central office personnel in Iran. If patients are not chosen for follow-up, their linkage data will be immediately destroyed. If they are chosen for follow-up, linkage data will be kept only as long as needed to complete follow-up and extraction of associated data from the TUCCP system. Only de-identified data sets will be used for analysis and no identifiable data will be sent to the US investigators. Access to data will be limited to those study personnel who need access for processing or analysis, and only after training in human subjects research and data safety procedures. Data sharing outside the study will be managed through mechanisms set out in the NIH document NOT-MH-14-015 (including the use of appropriate language describing data sharing in the consent process).

<u>4. Privacy for children/youth and parents:</u> The consent process will make it clear that the parent and child/youth are free to discuss the SDQ, GHQ, and other forms with the GP. Regardless, any concern for serious mental health problems with possible harm to self or others will be reported to the GP (including positive parent responses for the self-harm item on the GHQ). This will be clearly stated in the consent.

Our GP training will address sensitivity to parent privacy in the context of integrating child/youth and parent mental health care. In most cases the parents will have a pre-existing, separate clinical relationship with the GP which should provide an avenue for a confidential discussion. Research assistants administering the GHQ to parents, should they feel the need to notify the GP of thoughts about self-harm, will be instructed to never do so in the presence of the child. Parents will be told about such possible disclosures as part of the consent process. However, unless there seems to be an emergency, the research assistants will consult with the parent (and with the Iranian PI if desired) to make the disclosure to the GP in a way that is maximally respectful of the parent's wishes and any additional clinical issues that might be relevant.

GP training will also cover the importance of asking about substance use and exposure to intimate partner violence, but this would take place as part of the clinical encounter and be at the GPs' discretion. GPs will be trained in the appropriate procedures to follow should a concern for child maltreatment arise during a visit. As noted above, *currently in Iran there is no legal obligation for GPs or study personnel to report suspected child maltreatment to authorities. Reporting obligations only apply to children who have been hospitalized.* TUCCP consultants are capable of helping GPs review options for responding to concerns about maltreatment and other forms of family violence.

To manage risk of disclosure during follow-up, research assistants will use only means of contact provided by parents and youth at the time of enrollment. When making follow-up calls,

research assistants will identify themselves only as calling on behalf of the GP until they are certain that they are speaking to the enrolled parent and that he or she is able to speak in confidence. Participants will have the right to leave the study at any point including declining to participate in follow-up.

5. Privacy for GPs: All provider data will be treated as confidential and managed in the same manner as patient data. In written accounts of the data for publication or internal reports care will be taken to sufficiently disguise quotes or results that could because of “small cell” issues lead to the inadvertent identification of a participant. Measures taken will include careful editing of data to remove names of individuals and practices, altering exact ages, not naming precise neighborhoods, and when necessary creating composite descriptions or paraphrases of accounts.

6. Coercion and involuntary participation by children/youth and parents: Children/youth and parents will not be able to chose whether or not their GP is participating in the study, but the consent process will disclose that the GP is participating in a trial of training whose purpose is to enable the GP to treat some mental health problems him or herself in collaboration with the CMHC. Our planned consent process is consistent with the Ottawa Statement on the Ethical Design and Conduct of Clinical Trials (Taljaard 2013). First, our initial stakeholder consultation process, while not designed to seek community consent, has served to identify needed changes to the study protocol (such as having parents as our informants even for youth 11-15). Second, though it is not possible to seek prior consent from GPs’ patients prior to GPs receiving child mental health training, for the duration of our study we will be seeking consent from patients coming to see trained GPs. The consent process will make it clear that patients may choose to request an immediate referral to the CMHC or any other mental health resource if they prefer that to following the GP’s advice for treatment (which may or may not include referral). This would be the equivalent of care as it is presently being provided. For this reason and because of the vulnerabilities discussed above we will make sure that consent processes are private and unhurried, with ample time for questions and decision-making and clear explanation of the option to not participate or to withdraw at a later point.

GPs cannot be blinded to which patients are participating in the study since patients may chose to ask questions prompted by administration of the baseline instruments (SDQ, GHQ, etc.). However, patient participation in the study involves only activities related to evaluation – consent or lack of it does not necessarily represent a preference for one form of treatment or another.

7. Coercion and involuntary participation by GPs: Consent forms will emphasize the voluntary nature of participation and that neither ongoing participation in TUCCP nor ongoing employment are in any way contingent on participation in the study.

8. Risk of inferior treatment of child/youth or parent mental health problems: The study design attempts to balance response burden and possible benefit by encouraging parents and children/youth to discuss with their doctor any concerns raised by completing the SDQ and GHQ. Intervention GPs will be supported in their use of treatments which are drawn from the evidence-based literature and that have been judged to be acceptable in the Iranian context through stakeholder consultation during the study’s developmental phase. In addition, there are several mechanisms that should minimize the risk of inferior treatment:

- Throughout the study both control and intervention GPs will continue to be members of the TUCCP and have ready access to mental health consultation and referral for both

adults and children/youth. GPs will be free to refer any child for further evaluation or treatment at a CMHC regardless of study group assignment.

- In the Iranian system, while families can not directly access the CMHC without a GP referral, there are other mental health resources that they can access directly. However, there may be differences in cost depending on the family's insurance – referral to the CMHC by a GP as part of the TUCCP results in free treatment while self-referral elsewhere may be subject to fees of varying sizes. The consent process will remind parents and youth of these issues and that they may request a CMHC referral from their GP at any time.
- As noted in the background section, the TUCCP involves regular monitoring of patient status by CMHC personnel who review records and discuss care of all patients registered with the system whether they are referred or cared for by the GP. This review is required before GPs can receive top-up payments for follow-up care. Thus, payment will be denied for follow-up care judged to be inappropriate.
- Receptionists, who provide case management, constitute a second set of "eyes" interacting with families and assessing progress. Receptionists can trigger either return visits to the GP or a request to the GP to initiate a referral.
- The study will have its own data safety management processes (see below).

9. Increased work for GPs: GPs will always have the option to refer patients to the CMHC if they find that their practice does not have the time to manage newly detected child/youth or adult mental health problems. There may also be offsetting benefits of taking on mental health treatment: there could be a reduction in return visits for somatic presentations of undetected underlying mental health problems, and there may be reductions in the frequency of visits with unanticipated psychosocial problems that disrupt a practice's ability to keep to its schedule of appointments. In addition, practices will receive top-up payments for patients they are able to successfully follow and manage.

10: DSMP: As noted above, the TUCCP has safety and quality of care monitoring built into its standard operating procedures; it also includes incentives for participating providers to adequately follow-up treated patients. However, the study will have its own data monitoring plan operating in parallel. The US and Iranian PIs will be primarily responsible for this second plan. Incoming data will be cleaned and compiled on a regular basis and examined for unusual or unexpected patterns. The PIs will be assisted in this task by a four-person DSMB that includes both Iranian and US members. The present version of the protocol reflects feedback from the DSMB. The DSMB will have internet meetings before study initiation, during the study roll-out, and at its conclusion.

Site level research personnel will be thoroughly trained in good clinical practice, human subjects procedures, and the importance of being alert for and documenting adverse events, protocol deviations, and participant concerns. All of these will be immediately communicated to the in-country director and the PIs. Lists of possible expected and unexpected adverse events will be developed during the initial stakeholder phase of the study and incorporated into training for research assistants in Iran and the US.

Benefits

Patient participants may benefit by becoming more aware that their GP and/or her/his connections to mental health services are available for discussion and treatment of child and adult mental health concerns. Clinician participants may receive feedback or instruction as part of the study that will be of interest to them and that could add to their skill level.

The general public may benefit if the study's interventions prove to be practical and effective, since children's mental health services are currently difficult to obtain in Iran as in many other middle-income countries. These benefits seem reasonable in comparison to the relatively small risk of involuntary disclosure of information.

Payment and remuneration

As noted above, GPs will receive about $200 for participation in the two-day child mental health or control booster training and the trial. Families will receive a payment of about $2 to compensate them for any extra time required at their index visit when they provide consent and baseline data.

Costs

There are no direct costs associated with participation by patients. Patients asked to return to the GP for mental health treatment might have to pay for the GP visit whereas their treatment, had they been referred to the CMHC, would be provided without a charge. However, patients are likely to live closer to the GP office than to the CHMC meaning that they would not have to expend the cost of travel and the considerable time it could take to get to the CMHC, so costs are likely to be equivalent.

There are no costs to GPs for participation in the child mental health training, but there could be opportunity costs for participating if GPs were to be forgoing clinical income to attend.

I

Literature Cited

Aarons GA, Hurlburt, M, Horwitz SM. Advancing a conceptual model of evidence-based practice implementation in public service sectors. Administration and Policy in Mental Health, 2011;38:4–23.

Abedzadeh-Kalahroudi M, Razi E, Sehat M, Asadi-Lari M. Psychometric properties of the world health organization disability assessment schedule II -12 Item (WHODAS II) in trauma patients. Injury. 2016 May;47(5):1104-8.

Agborsangaya CB, Lahtinen M, Cooke T, Johnson JA. Comparing the EQ-5D 3L and 5L: measurement properties and association with chronic conditions and multimorbidity in the general population. Health Qual Life Outcomes. 2014 May 16;12:74.

Andrews G, Kemp A, Sunderland M, Von Korff M, Ustun TB. Normative data for the 12 item WHO Disability Assessment Schedule 2.0. PLoS One. 2009 Dec 17;4(12):e8343.

Asarnow JR, Jaycox LH, Duan N, LaBorde AP, Rea MM, Murray P, et al. Effectiveness of a quality improvement intervention for adolescent depression in primary care clinics: A randomized controlled trial. JAMA. 2005;293:311-9.

Chorpita BF, Daleiden EL, Weisz JR. Identifying and selecting the common elements of evidence based interventions: A distillation and matching model. Ment Health Serv Res. 2005;7:5-20.

Cross SPM, Hermens DF, Scott EM,Ottavio A, McGorry PD, Hickie IB. A clinical staging model for early intervention in youth mental health services. Psychiatric Services in Advance, May 15, 2014; doi: 10.1176/appi.ps.201300221

Cuijpers P, Van Straten A, Smit F. Preventing the incidence of new cases of mental disorders: a meta-analytic review. J Nerv Ment Dis. 2005 Feb;193(2):119-25.

Cuijpers P, van Straten A, Smit F, Mihalopoulos C, Beekman A. Preventing the onset of depressive disorders: a meta-analytic review of psychological interventions. Am J Psychiatry. 2008 Oct;165(10):1272-80.

Davis TS, Scheer SD, Gavazzi SM, Uppal R. Parent advocates in children's mental health: Program implementation processes and considerations. Administration and Policy in Mental Health. 2010:37(6):468-483.

Jerene D, Biru M, Teklu A, Rehman T, Ruff A, Wissow L. Factors promoting and inhibiting sustained impact of a mental health task-shifting program for HIV providers in Ethiopia. Global Mental Health (in press).

Katon W, Ünützer J, Wells K, Jones L. Collaborative depression care: History, evolution and ways to enhance dissemination and sustainability. General Hospital Psychiatry. 2010;32(5):456-464.

Katon W, Unützer J. Consultation psychiatry in the medical home and accountable care organizations: achieving the Triple Aim. Gen Hosp Psychiatry. 2011 ; 33(4): 305–310.

Khadivi R, Shakeri M, Ghobadi S. The Efficiency of Mental Health Integration in Primary Health Care: a Ten-year Study. Int J Prev Med. 2012 Mar;3(Suppl 1):S139-45.

Kiadaliri AA. A comparison of Iran and UK EQ-5D-3L value sets based on visual analogue scale. Int J Health Policy Mgmt 2017;6:267-272.

Kieling C, Baker-Henningham HB, Belfer M, Conti G, Ertem I, Omigbodun O, Rohde LA, Srinath S, Ulker N, Rahman A. Child and adolescent mental health worldwide: evidence for action. Lancet 2011;378:1515-25.

Kolko DJ, Campo J, Kilbourne AM, Hart J, Sakolsky D, Wisniewski S. Collaborative care outcomes for pediatric behavioral health problems: a cluster randomized trial. Pediatrics. 2014 Apr;133(4):e981-92.

Labhardt ND, Schiess K, Manga E, Langewitz W. Provider-patient interaction in rural Cameroon--how it relates to the patient's understanding of diagnosis and prescribed drugs, the patient's concept of illness, and access to therapy. Patient Educ Couns. 2009 Aug;76(2):196-201.

McGarry J, McNicholas F, Buckley H, Kelly BD, Atkin L, Ross N. The clinical effectiveness of a brief consultation and advisory approach compared to treatment as usual in child and adolescent mental health services. Clinical Child Psychology and Psychiatry. 2008;13(3), 365-376.

Moinfar Z, Sedaghat M, Abolhassani F et al. A collaborative care program for management of common mental disorders among diabetic patients in a primary healthcare setting. J Public Health 2016;24: 273.

Morgan DL. Focus groups as qualitative research. Newbury Park: Sage Publications, 1988.

Patel V, Kieling C, Maulik PK, Divan G. Improving access to care for children with mental disorders: a global perspective. Arch Dis Child 2013;98:323-327.

Perkins, R., & Scarlett, G. The effectiveness of single session therapy in child and adolescent mental health. part 2: An 18-month follow-up study. *Psychology and Psychotherapy.* 2008. *8*1(Pt 2), 143-156.

Scrimshaw SCM and Hurtado E. Rapid Assessment Procedures for Nutrition and Primary Health Care: Anthropological Approaches to Improving Programme Effectiveness. Los Angeles: UCLA Latin American Center, 1987.

Sharifi V. Community Mental Health Center. Tehran, 2011; Available from: http://cmhc.tums.ac.ir.

Sharifi V, Abolhasani F, Farhoudian A, Amin-Esmaeili M. Community Mental Health Centers in Iran: Planning Evidence-based Services . IJPCP. 2014; 19 (3) :163-176. URL: http://ijpcp.iums.ac.ir/article-1-2069-en.html.

Sharifi V, Aminesmaeili M, Hajebi A, Motevalian A, Rad-Goodarzi R, Hefazi M, Rahimi-Movaghar A. Twelve-month prevalence and correlates of psychiatric disorders in Iran: The Iran Mental Health Survey, 2011. Archives of Iranian Medicine. 2015; 18:2: 76-84

Wang PS, Berglund PA, et al. Delays in initial treatment contact after first onset of a mental disorder. Health Serv Res. 2004;39(2): 393-415.

Wang PS, Angermeyer M, et al. Delay and failure in treatment seeking after first onset of mental disorders in the World Health Organization's World Mental Health Survey Initiative. World Psychiatry 2007;6(3): 177-185.

Wissow LS, Gadomski A, Roter D, Larson S, Horn I, Bartlett E, Brown J, Zachary C, Luo X, Wang M-C. A cluster-randomized trial of mental health communication skills for pediatric generalists. Pediatrics 2008 Feb;121(2):266-75.

Wissow L, Gadomski A, Roter D, Larson S, Lewis B, Brown J. Aspects of mental health communication skills training that predict parent and child outcomes in pediatric primary care. Patient Educ Couns. 2011 Feb;82(2):226-32.

Wissow LS, Brown J, Fothergill K, Gadomski A, Hacker K, Salmon P, Zelkowitz. Mental health screening in pediatric primary care: a systematic review. J Am Acad Child Psychiatry 2013 Nov;52(11):1134-1147.e23.

Wissow LS, Tegegn T, Kassahun A, McNabb M, Tilahun T, Jerene D, Ruff A. Collaboratively re-framing mental health for integration with HIV care in Ethiopia. Health, Policy, and Planning 2014 Jul 10. pii: czu058.

World Health Organization (WHO). *Caring for children and adolescents with mental disorders: setting WHO directions*. Geneva, World Health Organization, 2003.

World Health Organization (WHO). Adolescent mental health: mapping actions of nongovernmental organizations and other international development organizations. Geneva, World Health Organization, 2012.

Yasamy MT, Bagheri Yazdi SA National Programme of Mental Health. Tehran: Ministry of Health and Medical Education, 2004

Original analysis section reproduced from published trial protocol

Data management

Incoming data from GP and patient instruments will be logged, examined for completeness, nonsense, and ambiguous entries. Queries will be sent to the research assistants to examine possibilities for re-contacting participants. Research assistants will use a messaging application to report on their daily progress with recruiting or follow-up and to be able to clarify or report any study issues immediately. Data collected on paper will be double entered. For each research assistant, a 5% sample of the participant forms that they collect will be checked by having a different member of the study team call the participant to verify key items. Supervisors of research assistants will make random visits to GP offices while research assistants are present to check on recruitment and data collection processes.

A trial data center in Tehran will be responsible for a) scoring baseline SDQ forms to determine which children/youth are eligible for the trial; b) maintaining files that link GPs to intervention status and children/youth to follow-up contact information; c) merging data collected directly from GPs and patients with data extracted from the TUCCP data system, and d) creating and maintaining unlinked merged data files. Duplicate merged unlinked files will be sent to the US study site for analyses, distribution to the Data Safety and Monitoring Board, and archiving. A final data set will be generated and deposited with the National Database for Clinical Trials Related to Mental Illness.

Data analysis approach

Analysis will follow a pre-determined plan and take place under the direction of the trial statistician. Analyses will be masked to study arm until data collection is finalized. Exploratory analyses will confirm expected distributions and the prevalence and patterns of missing data.

We will use appropriate methods for missing or censored data, such as multiple imputation or full information maximum likelihood [59].

The outcomes in each set of analyses will first be explored with simple bivariate statistics. Multilevel models with random effects will model patient outcomes, which are clustered within GPs [60]. The basic approach has the following form: if the study ultimately has *I* clusters (i=1:I), 3 time points (j=1:3), and *N* patients per cluster (k=1:N), individual patient outcomes are modeled as $Y_{ijk} = \mu_{ij} + \gamma C_{ijk} + e_{ijk}$ where e_{ijk} has a $^{iid}N(0,\sigma^2_e)$ distribution. Individual covariates included at this level (denoted by C) will include child age, gender, and initial SDQ symptom and functioning scores. The mean outcome for cluster i at time j can be expressed as: $\mu_{ij} = \mu + \alpha_i + \beta Z_{ij} + X_{ij}\theta$, where α_i is a random effect for the cluster and Z_{ij} *are the cluster-level covariates* (e.g., public or private GP practice, practice size, the three staggered training waves to which the GP belongs), and X_{ij} = 0 if the cluster is a control practice or = 1 if it is an intervention practice.

Interaction terms between intervention status and a hypothesized cluster-level moderator (e.g. GP's public or private practice, practice size, training wave) will be used for exploratory analyses of effect heterogeneity.

Analysis will be by intent-to-treat based on the intervention status of the GP. However, intent-to-treat analysis does not take into account the level of intervention actually received by individual patients. Therefore, the intent-to-treat data will be presented along with data describing, for each arm: 1) the proportion of patients receiving any form of collaborative mental health treatment, 2) the proportion of patients who do receive mental health treatment who receive it solely from a CMHC; and 3) the proportion of patients whose GP left the collaborative during the course of the study. Only (3) will be considered a protocol deviation, since per the

protocol, GPs may opt to treat or refer any patient. In addition, the impact of implementation outcomes such as GP changes in attitude and confidence (“post-treatment mediator” variables) will be examined using causal mediation methods [61].
