# Supplementary material for: Effect of General Practitioner Training in a Collaborative Child Mental Health Care Program on Children’s Mental Health Outcomes in a Low-Resource Setting: A Cluster Randomized Trial
Source: JAMA Psychiatry. 2022 Nov 30;80(1):22–30. doi: 10.1001/jamapsychiatry.2022.3989 (PMC9713683; doi:10.1001/jamapsychiatry.2022.3989)
Supplement: Supplement 2. — eAppendix 1. Data Analysis Plan and Sample Output eTable 1. Potential Confounders’ Relationship to Primary and Secondary Outcomes, Adjusted for Clustering With GP eTable 2. Crude SDQ Total Problems Scores by Time and Treatment Group, Adjusted Coefficients for Treatment Effect by Time eTable 3. Crude GHQ Total Scores by Time and Treatment Group, Adjusted Coefficients for Treatment Effect by Time eTable 4. Crude SDQ Total Problems Scores by Intervention Group, Time, and Child- or Adult-Predominant GP Practice, Adjusted Coefficient for Difference in Group-Time Interaction by Practice Type eTable 5. Baseline Factors Related to Children/Parents Being Lost to Follow-up at 6 Months eAppendix 2. Sensitivity Analyses: Alternative Approaches to Analysis [file jamapsychiatry-e223989-s002.pdf]

Supplemental Online Content

Sharifi V, Shahrivar Z, Zarafshan H, et al. Effect of general practitioner training in a collaborative child mental health care program on children's mental health outcomes in a low-resource setting: a cluster randomized trial. *JAMA Psychiatry.* Published online November 30, 2022. doi:10.1001/jamapsychiatry.2022.3989

eAppendix 1. Data Analysis Plan and Sample Output

eTable 1. Potential Confounders' Relationship to Primary and Secondary Outcomes, Adjusted for Clustering With GP

eTable 2. Crude SDQ Total Problems Scores by Time and Treatment Group, Adjusted Coefficients for Treatment Effect by Time

eTable 3. Crude GHQ Total Scores by Time and Treatment Group, Adjusted Coefficients for Treatment Effect by Time

eTable 4. Crude SDQ Total Problems Scores by Intervention Group, Time, and Child- or Adult-Predominant GP Practice, Adjusted Coefficient for Difference in Group-Time Interaction by Practice Type

eTable 5. Baseline Factors Related to Children/Parents Being Lost to Follow-up at 6 Months

eAppendix 2. Sensitivity Analyses: Alternative Approaches to Analysis

This supplemental material has been provided by the authors to give readers additional information about their work.

© 2022 Sharifi V et al. *JAMA Psychiatry.*

eAppendix 1. Data Analysis Plan and Sample Output

Detailed description of data analysis with examples

This section repeats information in the published trial protocol[1] and main manuscript but provides additional detail.

A: Overall

Analysis followed a pre-determined plan and took place under the direction of the trial statistician. Analyses were masked to study arm until data collection was finalized and initial univariate analyses were completed. These exploratory analyses confirmed expected distributions and the prevalence and patterns of missing data. For the primary outcome measures we used methods specific to the SDQ and GHQ to replace missing values.[2,3] Since only small numbers of binary baseline descriptive data were missing, we made calculations initially using listwise deletion of observations, and then conducted sensitivity analyses replacing missing values in different ways (all 0, all 1). None of these analyses changed the primary outcome.

B. Model for the main SDQ outcome

The main patient outcome was first explored with simple bivariate statistics. We planned to measure several baseline factors to characterize our sample, be able to adjust analyses in the case of unequal distribution between the intervention and control groups, and for further analyses to explore subgroups for which the intervention may be more or less effective. These include, for children/youth, family demographics (income, parental education, ethnicity, distance from treatment sites) and age and gender. While we did not expect that age and gender would vary between children/youth in the two study groups, it was possible that there would be differences in age or gender of children who actually receive treatment. We also examined, as planned, GP factors that could be related to implementation outcomes, including training, prior

© 2022 Sharifi V et al. *JAMA Psychiatry.*

activity in the collaborative care program, length of time in practice at current location, and proportion of children in the overall practice volume.

Multilevel models with random effects were then used to model the primary outcomes, which are clustered within GPs. The basic approach had the following form: if the study ultimately had *I* clusters (i=1:I), 3 time points (j=1:3), and *N* patients per cluster (k=1:N), individual patient outcomes are modeled as $Y_{ijk} = \mu_{ij} + \gamma C_{ijk} + e_{ijk}$ where e_{ijk} has a $^{iid} N(0,\sigma^2_e)$ distribution. Individual covariates at this level (denoted by C) included child and parent variables associated with the outcome in bivariate relationships. The mean outcome for cluster i at time j can be expressed as: $\mu_{ij} = \mu + \alpha_i + \beta Z_{ij} + X_{ij}\theta$, where α_i is a random effect for the cluster and Z_{ij} *are the cluster-level covariates* (e.g., public or private GP practice, practice size, the three staggered training waves to which the GP belongs), and X_{ij} = 0 if the cluster is a control practice or = 1 if it is an intervention practice. The Stata 16 SE code used in the final analyses is presented below (B1, B2).

Analysis was by intent-to-treat based on the intervention status of the GP. We did not conduct a per-protocol analysis, but do present the intent-to-treat data along with data describing, for each arm, whether the patient received mental health services during the course of the study.

Change to analysis plan: We had originally planned to present, alongside the intent-to-treat analysis: 1) the proportion of patients receiving any form of collaborative mental health treatment, 2) the proportion of patients who received mental health treatment who received it solely from a CMHC; and 3) the proportion of patients whose GP left the collaborative during the course of the study. The study was not able to assess (1) and (2) in the way that had been

© 2022 Sharifi V et al. *JAMA Psychiatry.*

initially planned. The study was only able to assess, by parent report, whether the patient had any mental health service “from a counselor, social worker, psychologist, or psychiatrist” at any location in the 6 months prior to or during the study (asked at baseline and 6 month observation period), whether the GP reported providing counseling, or whether the parent reported discussing a child psychosocial problem with the GP (See C1, below). Regarding (3), no GPs for whom patients were enrolled left the study. In addition, the plan to assess the impact of implementation outcomes such as GP changes in attitude and confidence (“post-treatment mediator” variables) will be reported in a separate paper.

1. Mixed effects multi-level regression model including only variables used in the non-factorial balanced allocation of GPs to treatment groups

Groupbin: treatment allocation, 1=intervention

Gpnumberreal: GP identifier

Code: child identifier

Timepoint (0=baseline, 1=3 months, 2=6 months)

Wave: study wave (1,2,3)

Publicsite: GP practice public (1) or private (0)

Caseloadbin: large (1) versus small (0) practice

```
mixed sdqtotprobs i.groupbin##i.timepoint publicsite caseloadbin i.Wave ||
gpnumberreal: || Code:, mle

Performing EM optimization:

Performing gradient-based optimization:

Iteration 0:   log likelihood = -3027.2791
Iteration 1:   log likelihood =  -3027.279

Computing standard errors:

Mixed-effects ML regression                     Number of obs     =      1,017
```

© 2022 Sharifi V et al. *JAMA Psychiatry.*

```
-------------------------------------------------------------
                |     No. of       Observations per Group
 Group Variable |     Groups    Minimum    Average    Maximum
----------------+--------------------------------------------
   gpnumberreal |         49          1       20.8         51
           Code |        389          1        2.6          3
-------------------------------------------------------------

                                                Wald chi2(9)      =     225.09
Log likelihood =  -3027.279                     Prob > chi2       =     0.0000

------------------------------------------------------------------------------------
       sdqtotprobs |      Coef.   Std. Err.      z    P>|z|     [95% Conf. Interval]
-------------------+----------------------------------------------------------------
        1.groupbin |  -.1441795   .7464233    -0.19   0.847    -1.607142    1.318783
                   |
         timepoint |
                1  |  -3.499696   .4281098    -8.17   0.000    -4.338776   -2.660617
                2  |  -4.513255   .4480864   -10.07   0.000    -5.391488   -3.635022
                   |
groupbin#timepoint |
              1 1  |    1.09491   .5827984     1.88   0.060     -.047354    2.237174
              1 2  |   .4524018   .6054431     0.75   0.455    -.7342448    1.639048
                   |
        publicsite |  -.1035253   .8854488    -0.12   0.907    -1.838973    1.631922
       caseloadbin |    .598699   .6891145     0.87   0.385    -.7519407    1.949339
                   |
              Wave |
                2  |   .4837923   .8388916     0.58   0.564    -1.160405     2.12799
                3  |  -1.194798   .9605448    -1.24   0.214    -3.077431    .6878349
                   |
             _cons |   19.93744   .8140875    24.49   0.000     18.34186    21.53303
------------------------------------------------------------------------------------

------------------------------------------------------------------------------
  Random-effects Parameters  |   Estimate   Std. Err.     [95% Conf. Interval]
-----------------------------+------------------------------------------------
gpnumberreal: Identity       |
                  var(_cons) |   3.022229    1.12845      1.453794    6.282781
-----------------------------+------------------------------------------------
```

© 2022 Sharifi V et al. *JAMA Psychiatry.*

```
Code: Identity               |
                  var(_cons) |    10.4834    1.26275      8.278909    13.27491
-----------------------------+------------------------------------------------
               var(Residual) |   14.55022    .809038      13.04789    16.22554
------------------------------------------------------------------------------
LR test vs. linear model: chi2(2) = 230.46                Prob > chi2 = 0.0000

Note: LR test is conservative and provided only for reference.

. lincom 1.groupbin + 1.groupbin#1.timepoint

 ( 1)  [sdqtotprobs]1.groupbin + [sdqtotprobs]1.groupbin#1.timepoint = 0

------------------------------------------------------------------------------
 sdqtotprobs |      Coef.   Std. Err.      z    P>|z|     [95% Conf. Interval]
-------------+----------------------------------------------------------------
         (1) |   .9507304   .7704898     1.23   0.217    -.5594018    2.460863
------------------------------------------------------------------------------

. lincom 1.groupbin + 1.groupbin#2.timepoint

 ( 1)  [sdqtotprobs]1.groupbin + [sdqtotprobs]1.groupbin#2.timepoint = 0

------------------------------------------------------------------------------
 sdqtotprobs |      Coef.   Std. Err.      z    P>|z|     [95% Conf. Interval]
-------------+----------------------------------------------------------------
         (1) |   .3082223   .7876748     0.39   0.696    -1.235592    1.852037
------------------------------------------------------------------------------
```

2. Intraclass correlation for baseline SDQ scores within GP

```
. mixed sdqtotprobs i.groupbin publicsite caseloadbin i.Wave if timepoint==0 ||
gpnumberreal:, mle

Performing EM optimization:

Performing gradient-based optimization:

Iteration 0:   log likelihood =  -1074.652
```

© 2022 Sharifi V et al. *JAMA Psychiatry.*

```
Iteration 1:   log likelihood = -1074.5532
Iteration 2:   log likelihood = -1074.5529
Iteration 3:   log likelihood = -1074.5529

Computing standard errors:

Mixed-effects ML regression                     Number of obs     =        388
Group variable: gpnumberreal                    Number of groups  =         49

                                                Obs per group:
                                                              min =          1
                                                              avg =        7.9
                                                              max =         20

                                                Wald chi2(5)      =       6.69
Log likelihood = -1074.5529                     Prob > chi2       =     0.2447

------------------------------------------------------------------------------
 sdqtotprobs |      Coef.   Std. Err.      z    P>|z|     [95% Conf. Interval]
-------------+----------------------------------------------------------------
  1.groupbin |   .0413239   .4461583     0.09   0.926    -.8331303    .9157782
  publicsite |  -.1503624   .5571686    -0.27   0.787    -1.242393    .9416679
 caseloadbin |    .850185   .4529904     1.88   0.061    -.0376598     1.73803
             |
        Wave |
          2  |   .0552446   .5550228     0.10   0.921     -1.03258    1.143069
          3  |  -.8254772   .6383728    -1.29   0.196    -2.076665    .4257105
             |
       _cons |    19.7584   .5371511    36.78   0.000      18.7056     20.8112
------------------------------------------------------------------------------

------------------------------------------------------------------------------
  Random-effects Parameters  |   Estimate   Std. Err.     [95% Conf. Interval]
-----------------------------+------------------------------------------------
gpnumberreal: Identity       |
                  var(_cons) |   .4461571   .4863367      .0526781    3.778731
-----------------------------+------------------------------------------------
               var(Residual) |   14.50271   1.107845      12.48611    16.84502
------------------------------------------------------------------------------
LR test vs. linear model: chibar2(01) = 1.14          Prob >= chibar2 = 0.1427
```

© 2022 Sharifi V et al. *JAMA Psychiatry.*

```
. estat icc

Residual intraclass correlation

------------------------------------------------------------------------------
                       Level |        ICC   Std. Err.     [95% Conf. Interval]
-----------------------------+------------------------------------------------
                gpnumberreal |   .0298455   .0322492      .0034553    .2144253
------------------------------------------------------------------------------
```

3. Mixed effects multi-level regression model including variables used in the non-factorial balanced allocation of GPs to treatment groups (same as above in 1) PLUS variables found in bivariate analyses to be associated with change in SDQ scores:

GHQpos0: parent GHQ positive at baseline
Childage11plus: child 11 years old or greater
GPatsite10plus: GP in practice at site 10 years or more
Gpgenderbin: GP female (1), male (0).

```
. mixed sdqtotprobs i.groupbin##i.timepoint publicsite caseloadbin i.Wave ghqpos0i
childage11plus gpatsite10plusnomiss gpgenderbin || gpnumberreal: || Code:, mle

Performing EM optimization:

Performing gradient-based optimization:

Iteration 0:   log likelihood = -3020.7311
Iteration 1:   log likelihood = -3020.7309

Computing standard errors:

Mixed-effects ML regression                     Number of obs     =      1,017

-------------------------------------------------------------
                |     No. of       Observations per Group
 Group Variable |     Groups    Minimum    Average    Maximum
----------------+--------------------------------------------
   gpnumberreal |         49          1       20.8         51
           Code |        389          1        2.6          3
-------------------------------------------------------------

                                                Wald chi2(13)     =     238.25
Log likelihood = -3020.7309                     Prob > chi2       =     0.0000

------------------------------------------------------------------------------------
         sdqtotprobs |      Coef.   Std. Err.      z    P>|z|     [95% Conf. Interval]
---------------------+--------------------------------------------------------------
          1.groupbin |   -.529357   .8160712    -0.65   0.517    -2.128827    1.070113
                     |
           timepoint |
                  1  |  -3.516468   .4280213    -8.22   0.000    -4.355374   -2.677561
                  2  |  -4.527431    .447921   -10.11   0.000    -5.405341   -3.649522
                     |
```

© 2022 Sharifi V et al. *JAMA Psychiatry.*

```
  groupbin#timepoint |
                 1 1  |   1.102596   .5826318     1.89   0.058    -.0393413    2.244534
                 1 2  |   .4466589    .605183     0.74   0.460    -.7394779    1.632796
                      |
          publicsite |  -.8080776   1.182691    -0.68   0.494    -3.126109    1.509954
         caseloadbin |   .9022639     .78138     1.15   0.248    -.6292128    2.433741
                      |
                Wave |
                   2  |   .2725737   .9226442     0.30   0.768    -1.535776    2.080923
                   3  |  -.8263664   1.072221    -0.77   0.441    -2.927881    1.275148
                      |
             ghqpos0i |   1.505577   .4252191     3.54   0.000     .6721624    2.338991
      childage11plus |   .2110415   .4640437     0.45   0.649    -.6984674     1.12055
gpatsite10plusnomiss |  -.8048113   1.073861    -0.75   0.454     -2.90954    1.299918
         gpgenderbin |   .5105081   .9154509     0.56   0.577    -1.283743    2.304759
               _cons |   19.51067   1.325868    14.72   0.000     16.91202    22.10933
--------------------------------------------------------------------------------------

------------------------------------------------------------------------------
  Random-effects Parameters  |   Estimate   Std. Err.     [95% Conf. Interval]
-----------------------------+------------------------------------------------
gpnumberreal: Identity       |
                  var(_cons) |   3.413954   1.244938      1.670541     6.97683
-----------------------------+------------------------------------------------
Code: Identity               |
                  var(_cons) |   9.751574   1.217565      7.634755     12.4553
-----------------------------+------------------------------------------------
               var(Residual) |   14.55174   .8090674      13.04935    16.22711
------------------------------------------------------------------------------
LR test vs. linear model: chi2(2) = 225.26                Prob > chi2 = 0.0000

Note: LR test is conservative and provided only for reference.

. lincom 1.groupbin + 1.groupbin#1.timepoint

 ( 1)  [sdqtotprobs]1.groupbin + [sdqtotprobs]1.groupbin#1.timepoint = 0

------------------------------------------------------------------------------
 sdqtotprobs |      Coef.   Std. Err.      z    P>|z|     [95% Conf. Interval]
-------------+----------------------------------------------------------------
         (1) |   .5732392    .838894     0.68   0.494    -1.070963    2.217441
------------------------------------------------------------------------------

. lincom 1.groupbin + 1.groupbin#2.timepoint

 ( 1)  [sdqtotprobs]1.groupbin + [sdqtotprobs]1.groupbin#2.timepoint = 0

------------------------------------------------------------------------------
 sdqtotprobs |      Coef.   Std. Err.      z    P>|z|     [95% Conf. Interval]
-------------+----------------------------------------------------------------
         (1) |  -.0826981    .856055    -0.10   0.923    -1.760535    1.595139
------------------------------------------------------------------------------
```

C. Secondary outcomes

1. . Model for the main secondary outcome (receipt of services)
seenpast6months (did child get MH services in the past six months) measured at baseline and then at 6 months (not in between – only two timepoints). Model has the variables used in non-factorial balanced allocation.

© 2022 Sharifi V et al. *JAMA Psychiatry.*

```
. melogit seenmhpast6mos i.groupbin##i.timepoint publicsite caseloadbin i.wavebin if
(timepoint==0 | timepoint==2) || gpnumberreal:  || Code:, or

Fitting fixed-effects model:

Iteration 0:   log likelihood = -353.53023
Iteration 1:   log likelihood =  -353.1208
Iteration 2:   log likelihood = -353.12064
Iteration 3:   log likelihood = -353.12064

Refining starting values:

Grid node 0:   log likelihood =   -349.228

Fitting full model:

Iteration 0:   log likelihood =   -349.228  (not concave)
Iteration 1:   log likelihood = -345.77111
Iteration 2:   log likelihood = -342.91654
Iteration 3:   log likelihood = -339.02868
Iteration 4:   log likelihood = -338.51818
Iteration 5:   log likelihood = -338.51178
Iteration 6:   log likelihood = -338.51176

Mixed-effects logistic regression               Number of obs     =        676

-------------------------------------------------------------
                |     No. of       Observations per Group
 Group Variable |     Groups    Minimum    Average    Maximum
----------------+--------------------------------------------
   gpnumberreal |         48          1       14.1         35
           Code |        380          1        1.8          2
-------------------------------------------------------------

Integration method: mvaghermite                 Integration pts.  =          7

                                                Wald chi2(7)      =      25.79
Log likelihood = -338.51176                     Prob > chi2       =     0.0005
------------------------------------------------------------------------------------
    seenmhpast6mos | Odds Ratio   Std. Err.      z    P>|z|     [95% Conf. Interval]
-------------------+----------------------------------------------------------------
        1.groupbin |   .8173302   .3339337    -0.49   0.622     .3669654    1.820413
       2.timepoint |   1.594389   .5704285     1.30   0.192     .7907837    3.214628
                   |
groupbin#timepoint |
              1 2  |   2.967079    1.43784     2.24   0.025     1.147727    7.670425
                   |
        publicsite |   .7003347   .2915582    -0.86   0.392     .3096989    1.583695
       caseloadbin |   .5366115   .1827359    -1.83   0.068     .2752927    1.045985
                   |
           wavebin |
                1  |   .8846349   .3608058    -0.30   0.764     .3977355    1.967586
                2  |    .639115   .3048778    -0.94   0.348     .2509165    1.627904
                   |
             _cons |   .1850099   .0851409    -3.67   0.000     .0750715    .4559477
-------------------+----------------------------------------------------------------
gpnumberreal       |
         var(_cons)|   .0560852   .2728075                      4.06e-06    774.8764
-------------------+----------------------------------------------------------------
gpnumberreal>Code  |
         var(_cons)|   3.709005   1.369108                      1.799089     7.64649
------------------------------------------------------------------------------------
Note: Estimates are transformed only in the first equation.
Note: _cons estimates baseline odds (conditional on zero random effects).
LR test vs. logistic model: chi2(2) = 29.22             Prob > chi2 = 0.0000

Note: LR test is conservative and provided only for reference.

. testparm 1.groupbin#2.timepoint
```

© 2022 Sharifi V et al. *JAMA Psychiatry.*

```
 ( 1)  [seenmhpast6mos]1.groupbin#2.timepoint = 0

          chi2(  1) =    5.04
        Prob > chi2 =    0.0248


. margins groupbin#timepoint

Predictive margins                              Number of obs     =        676
Model VCE    : OIM

Expression   : Marginal predicted mean, predict()

------------------------------------------------------------------------------------
                  |            Delta-method
                  |     Margin   Std. Err.      z    P>|z|     [95% Conf. Interval]
------------------+-----------------------------------------------------------------
groupbin#timepoint |
              0 0  |   .1888877    .030463     6.20   0.000     .1291814     .248594
              0 2  |   .2397604   .0360118     6.66   0.000     .1691786    .3103421
              1 0  |   .1691116   .0261745     6.46   0.000     .1178104    .2204127
              1 2  |   .3543216   .0375788     9.43   0.000     .2806686    .4279747
------------------------------------------------------------------------------------
```

2. Model for other secondary outcomes that are measured only at one time point (the parent or GP reports of what happened at the index visit); includes variables used in treatment allocation

```
. melogit disanyparprobbin i.groupbin publicsite caseloadbin i.wavebin if timepoint==0 ||
gpnumberreal:, or

Fitting fixed-effects model:

Iteration 0:   log likelihood = -249.04643
Iteration 1:   log likelihood = -248.72695
Iteration 2:   log likelihood = -248.72671
Iteration 3:   log likelihood = -248.72671

Refining starting values:

Grid node 0:   log likelihood = -243.35885

Fitting full model:

Iteration 0:   log likelihood = -243.35885
Iteration 1:   log likelihood = -242.58419
Iteration 2:   log likelihood = -242.55618
Iteration 3:   log likelihood = -242.55616
Iteration 4:   log likelihood = -242.55616

Mixed-effects logistic regression               Number of obs     =        382
Group variable:    gpnumberreal                 Number of groups  =         48

                                                Obs per group:
                                                              min =          1
                                                              avg =        8.0
                                                              max =         20

Integration method: mvaghermite                 Integration pts.  =          7

                                                Wald chi2(5)      =       9.90
Log likelihood = -242.55616                     Prob > chi2       =     0.0781
----------------------------------------------------------------------------------
disanyparprobbin | Odds Ratio   Std. Err.      z    P>|z|     [95% Conf. Interval]
-----------------+----------------------------------------------------------------
```

© 2022 Sharifi V et al. *JAMA Psychiatry.*

```
      1.groupbin |   2.265199   .7686668     2.41   0.016     1.164828    4.405051
      publicsite |   .4037353   .1745738    -2.10   0.036     .1729976    .9422224
     caseloadbin |   1.134517   .3869216     0.37   0.711     .5814501    2.213651
                 |
         wavebin |
              1  |   1.175113   .4869709     0.39   0.697     .5216018    2.647406
              2  |    1.22916   .5841054     0.43   0.664     .4842964    3.119646
                 |
           _cons |   .9436347   .3707685    -0.15   0.883     .4368716    2.038234
-----------------+----------------------------------------------------------------
gpnumberreal     |
       var(_cons)|   .5963205     .29652                      .2250196    1.580299
----------------------------------------------------------------------------------
Note: Estimates are transformed only in the first equation.
Note: _cons estimates baseline odds (conditional on zero random effects).
LR test vs. logistic model: chibar2(01) = 12.34       Prob >= chibar2 = 0.0002
```

C. Subgroup analysis for GPs who have child-predominant practices)

As planned, interaction terms between intervention status and hypothesized cluster-level moderator (e.g. GP's public or private practice, practice size, training wave) were used for exploratory analyses of effect heterogeneity.

All variables same as above plus:

Gpseesmore1: (1) if GP reports that their practice is composed 50% or more of children

```
. mixed sdqtotprobs i.groupbin##i.timepoint##i.gpseesmore1 publicsite caseloadbin i.Wave
childage11plus gpatsite10plusnomiss gpgenderbin ghqpos0i || gpnumberreal: || Code:, mle

Performing EM optimization:

Performing gradient-based optimization:

Iteration 0:   log likelihood = -3006.7727
Iteration 1:   log likelihood = -3006.7726

Computing standard errors:

Mixed-effects ML regression                     Number of obs     =      1,017

-------------------------------------------------------------
                |     No. of       Observations per Group
 Group Variable |     Groups    Minimum    Average    Maximum
----------------+--------------------------------------------
   gpnumberreal |         49          1       20.8         51
           Code |        389          1        2.6          3
-------------------------------------------------------------

                                                Wald chi2(19)     =     276.70
Log likelihood = -3006.7726                     Prob > chi2       =     0.0000

------------------------------------------------------------------------------------------
                  sdqtotprobs |      Coef.   Std. Err.      z    P>|z|     [95% Conf. Interval]
------------------------------+-----------------------------------------------------------
                   1.groupbin |  -.8258893   1.030356    -0.80   0.423    -2.845351    1.193572
                              |
                    timepoint |
                           1  |  -3.598961   .6307479    -5.71   0.000    -4.835204   -2.362718
                           2  |  -5.200679    .663281    -7.84   0.000    -6.500686   -3.900672
                              |
           groupbin#timepoint |
                         1 1  |   2.066658   .7871601     2.63   0.009     .5238523    3.609463
                         1 2  |   2.051747   .8228764     2.49   0.013     .4389387    3.664555
                              |
                1.gpseesmore1 |  -1.762107   1.041918    -1.69   0.091    -3.804229    .2800146
                              |
         groupbin#gpseesmore1 |
```

© 2022 Sharifi V et al. *JAMA Psychiatry.*

```
                         1 1  |   .4900907   1.537731     0.32   0.750    -2.523807    3.503988
                              |
        timepoint#gpseesmore1 |
                         1 1  |   .1624891   .8485855     0.19   0.848    -1.500708    1.825686
                         2 1  |   1.212074   .8890787     1.36   0.173    -.5304877    2.954637
                              |
groupbin#timepoint#gpseesmore1 |
                       1 1 1  |  -2.984977   1.193978    -2.50   0.012    -5.325129   -.6448236
                       1 2 1  |  -4.132628   1.236714    -3.34   0.001    -6.556543   -1.708712
                              |
                   publicsite |  -.3596589   1.018744    -0.35   0.724     -2.35636    1.637042
                  caseloadbin |   .7627824   .7109757     1.07   0.283    -.6307043    2.156269
                              |
                         Wave |
                           2  |   .0457372   .8085861     0.06   0.955    -1.539062    1.630537
                           3  |  -1.325999   .9857905    -1.35   0.179    -3.258113     .606115
                              |
               childage11plus |   .1613602   .4636165     0.35   0.728    -.7473114    1.070032
         gpatsite10plusnomiss |  -.4874344   .9343733    -0.52   0.602    -2.318772    1.343904
                  gpgenderbin |    .056935   .8467002     0.07   0.946    -1.602567    1.716437
                      ghqpos0i |   1.495255   .4242809     3.52   0.000     .6636795     2.32683
                        _cons |   20.49122   1.235062    16.59   0.000     18.07054     22.9119
------------------------------------------------------------------------------------------------

------------------------------------------------------------------------------
  Random-effects Parameters  |   Estimate   Std. Err.     [95% Conf. Interval]
-----------------------------+------------------------------------------------
gpnumberreal: Identity       |
                  var(_cons) |   2.040928   .9618644      .8103392    5.140301
-----------------------------+------------------------------------------------
Code: Identity               |
                  var(_cons) |   10.05486   1.232036      7.908193    12.78424
-----------------------------+------------------------------------------------
               var(Residual) |   14.13906   .7863192      12.67892    15.76734
------------------------------------------------------------------------------
LR test vs. linear model: chi2(2) = 198.46                Prob > chi2 = 0.0000

Note: LR test is conservative and provided only for reference.

. . lincom 1.groupbin#1.gpseesmore1

 ( 1)  [sdqtotprobs]1.groupbin#1.gpseesmore1 = 0

------------------------------------------------------------------------------
 sdqtotprobs |      Coef.   Std. Err.      z    P>|z|     [95% Conf. Interval]
-------------+----------------------------------------------------------------
         (1) |   .4900907   1.537731     0.32   0.750    -2.523807    3.503988
------------------------------------------------------------------------------

. lincom 1.groupbin#1.gpseesmore1+1.groupbin#1.timepoint#1.gpseesmore1

 ( 1)  [sdqtotprobs]1.groupbin#1.gpseesmore1 + [sdqtotprobs]1.groupbin#1.timepoint#1.gpseesmore1
= 0

------------------------------------------------------------------------------
 sdqtotprobs |      Coef.   Std. Err.      z    P>|z|     [95% Conf. Interval]
-------------+----------------------------------------------------------------
         (1) |  -2.494886   1.598837    -1.56   0.119    -5.628549     .638777
------------------------------------------------------------------------------

. lincom 1.groupbin#1.gpseesmore1+1.groupbin#2.timepoint#1.gpseesmore1

 ( 1)  [sdqtotprobs]1.groupbin#1.gpseesmore1 + [sdqtotprobs]1.groupbin#2.timepoint#1.gpseesmore1
= 0

------------------------------------------------------------------------------
 sdqtotprobs |      Coef.   Std. Err.      z    P>|z|     [95% Conf. Interval]
-------------+----------------------------------------------------------------
         (1) |  -3.642537   1.631548    -2.23   0.026    -6.840313   -.4447613
------------------------------------------------------------------------------
```

© 2022 Sharifi V et al. *JAMA Psychiatry.*

Citations

1. Sharifi V, Shahrivar Z, Zarafshan H, et al. Collaborative care for child and youth mental health problems in a middle-income country: study protocol for a randomized controlled trial training general practitioners. *Trials.* 2019;20(1):405.

2. Scoring the SDQ. https://sdqinfo.org/py/sdqinfo/c0.py. Accessed July 8, 2021.

3. Hjelle EG, Bragstad LK, Zucknick M, Kirkevold M, Thommessen B, Sveen U. The General Health Questionnaire-28 (GHQ-28) as an outcome measurement in a randomized controlled trial in a Norwegian stroke population. *BMC Psychol.* 2019;7(1):18.

© 2022 Sharifi V et al. *JAMA Psychiatry.*

eTable 1. Potential Confounders' Relationship to Primary and Secondary Outcomes, Adjusted for Clustering With GP

Potential confounder	Change in SDQ total problems score (95% CI)*	Change in GHQ total score (95% CI)*
Child/parent characteristics		
Child age 11+ versus 5-10 years	**-1.7 (-3.0, -0.42)**	.49 (-2.8, 3.8)
Child female versus male	-.44 (-1.6, 0.74)	-2.3 (-5.3, 0.78)
Parent GHQ positive at baseline	1.2 (-0.05, 2.4)	**-11.2 (-14.0, -8.4)**
Family does not have enough money for bills	.05 (-1.2, 1.3)	.12 (-3.0, 3.2)
Child's first visit with this GP	-.77 (-2.3, 0.76)	2.7 (-1.1, 6.5)
GP characteristics		
Larger practice size	-1.0 (-2.8, 0.77)	2.1 (-1.4, 5.5)
Public practice sites	.73 (-1.3, 2.8)	**4.1 (0.59, 7.5)**
GP gender female versus male	**1.8 (0.07, 3.5)**	.06 (-3.4, 3.5)
GP at site 10 years or more	**-2.3 (-4.0, -0.63)**	**-4.3 (-7.5, -1.2)**
GP practice predominantly children (half or more of patients)	-.98 (-2.8, 0.84)	**-3.3 (-6.7, 0.01)**

* Bold face results are significant at a level of p<.05 using two-tailed tests

Abbreviations: SDQ, Strengths and Difficulties Questionnaire; GHQ, General Health Questionnaire, GP, General Practitioner

eTable 2. Crude SDQ Total Problems Scores by Time and Treatment Group, Adjusted Coefficients for Treatment Effect by Time

	Intervention		Control		
	N	Mean (SD)	N	Mean (SD)	Intervention v. Control (95% CI)[a]
Baseline	215[b]	20.1 (3.8)	173	20.0 (4.0)	-0.53 (-2.13 to 1.07)
3 months	177	17.8 (6.0)	154	16.4 (6.2)	0.57 (-1.07 to 2.22)
6 months	163	16.0 (6.3)	135	15.4 (6.3)	-.08 (-1.76 to 1.59)

Abbreviation: SDQ, Strengths and Difficulties Questionnaire

[a] 3 and 6-month intervention v. control comparisons are linear combinations of group and group*time coefficients

[b] Total number 216; one patient was identified as positive on the SDQ at baseline but the actual score was subsequently lost. Scores for this patient were available at 3 and 6 months.

© 2022 Sharifi V et al. *JAMA Psychiatry.*

eTable 3. Crude GHQ Total Scores by Time and Treatment Group, Adjusted Coefficients for Treatment Effect by Time

	Intervention		Control		
	N	Mean (SD)	N	Mean (SD)	Intervention v. Control (95% CI)[a]
Baseline	210	29.1 (13.7)	161	29.1 (15.1)	-1.0 (-3.7, 1.6)
3 months	173	24.1 (13.8)	150	25.2 (12.4)	-2.1 (-4.9, 0.67)
6 months	161	24.1 (13.1)	133	24.2 (14.2)	-1.6 (-4.5, 1.3)

Abbreviations: GHQ, General Health Questionnaire; GP, General Practitioner

[a] adjusted for clustering within GP plus other variables associated with change in GHQ scores: parent GHQ status at baseline (positive vs. negative), GP's length of time in practice, GP practice composition (child or adult-predominant)) and the variables used to balance randomization

© 2022 Sharifi V et al. *JAMA Psychiatry.*

eTable 4. Crude SDQ Total Problems Scores by Intervention Group, Time, and Child- or Adult-Predominant GP Practice, Adjusted Coefficient for Difference in Group-Time Interaction by Practice Type

	Children in child-predominant practices					Children in adult-predominant practices					Adjusted coefficient comparing across practice types (95% CI) [b]
	Intervention		Control			Intervention		Control			
	N	Mean (SD)	N	Mean (SD)	Effect estimate for intervention (95% CI)[a]	N	Mean (SD)	N	Mean (SD)	Effect estimate for intervention (95% CI) [a]	
Baseline	68	19.1 (3.6)	92	19.2 (3.4)	-.43 (-2.3, 1.4)	137	20.4 (3.8)	81	20.9 (4.5)	-1.0 (-3.1, 1.2)	.58 (-2.6, 3.7)
3 months	56	15.0 (5.6)	86	15.7 (5.6)	-1.3 (-3.2, 0.64)	113	18.9 (5.8)	68	17.4 (6.7)	1.1 (-1.2, 3.3)	-2.4 (-5.7, 0.82)
6 months	53	13.2 (5.6)	76	15.1 (5.5)	-2.5 (-4.5, 0.52)	103	17.2 (6.1)	59	15.8 (7.2)	1.1 (-0.13, 3.4)	-3.5 (-6.9, -0.21)

Abbreviations: SDQ, Strengths and Difficulties Questionnaire; GP, General Practitioner

[a] Adjusted for variables used in balanced random allocation, study wave, parent GHQ positivity at baseline, child age, GP length of time at site and GP gender

[b] Coefficient represents test of three-way interaction of intervention group, time, and practice type for difference between intervention and control SDQ total problems score, adjusted for variables used in balanced random allocation, study wave, parent GHQ positivity at baseline, child age, GP length of time at site and GP gender.

© 2022 Sharifi V et al. *JAMA Psychiatry.*

eTable 5. Baseline Factors Related to Children/Parents Being Lost to Follow-up at 6 Months

Variable	Odds of being lost to follow-up at 6 months observation[a]	95% confidence limits on odds of being lost to follow-up
Cared for by treatment versus control GP	1.15	.62, 2.15
Cared for by GP at public versus private site	1.03	.54, 1.94
Cared for by female versus male GP	.73	.42, 1.28
Cared for by GP at large versus small site	.84	.47, 1.51
Cared for by GP at site 10 years or more versus less than 10 years	1.76	1.04, 3.01
Care for by GP in child-predominant versus adult-predominant practice	.72	.40, 1.27

[a] bivariate relationships between variable and child data missing at 6 months observation, adjusted for clustering within GPs

© 2022 Sharifi V et al. *JAMA Psychiatry.*

eAppendix 2. Sensitivity Analyses: Alternative Approaches to Analysis

Sensitivity analysis 1: examining main and sub-group outcomes using post-pre differences per patient

a. Main outcome modeled as change in SDQ total problems score at 6 months minus baseline score

```
. mixed deltasdq sdqtotprobs0 groupbin wavebin publicsite caseload || gpnumberreal:

Performing EM optimization:

Performing gradient-based optimization:

Iteration 0:   log likelihood =  -910.1881
Iteration 1:   log likelihood =  -910.1881

Computing standard errors:

Mixed-effects ML regression                     Number of obs     =        297
Group variable: gpnumberreal                    Number of groups  =         45

                                                Obs per group:
                                                              min =          1
                                                              avg =        6.6
                                                              max =         15

                                                Wald chi2(5)      =      13.47
Log likelihood =  -910.1881                     Prob > chi2       =     0.0193

------------------------------------------------------------------------------
    deltasdq |      Coef.   Std. Err.      z    P>|z|     [95% Conf. Interval]
-------------+----------------------------------------------------------------
sdqtotprobs0 |  -.2522371   .0765523    -3.29   0.001    -.4022768   -.1021974
    groupbin |   .3265354   .9013719     0.36   0.717    -1.440121    2.093192
     wavebin |  -.6971286   .6449605    -1.08   0.280    -1.961228    .5669709
  publicsite |   1.025396   1.157387     0.89   0.376    -1.243041    3.293833
    caseload |   .8192557   .9199263     0.89   0.373    -.9837668    2.622278
       _cons |   .5949644   1.845248     0.32   0.747    -3.021655    4.211584
------------------------------------------------------------------------------

------------------------------------------------------------------------------
  Random-effects Parameters  |   Estimate   Std. Err.     [95% Conf. Interval]
-----------------------------+------------------------------------------------
gpnumberreal: Identity       |
                  var(_cons) |   4.747011   1.815574      2.243179    10.04562
-----------------------------+------------------------------------------------
               var(Residual) |   23.84542   2.100633      20.06408    28.33939
------------------------------------------------------------------------------
LR test vs. linear model: chibar2(01) = 18.64         Prob >= chibar2 = 0.0000
```

b. Subgroup (GP practice child versus adult predominant) modeled as change in SDQ total problems score at 6 months minus baseline score

```
. mixed deltasdq sdqtotprobs0 i.groupbin##i.gpseesmorekids wavebin publicsite
caseload|| gpnumberreal:

Performing EM optimization:

Performing gradient-based optimization:

Iteration 0:   log likelihood = -883.77506
Iteration 1:   log likelihood = -883.77463
```

© 2022 Sharifi V et al. *JAMA Psychiatry.*

```
Iteration 2:   log likelihood = -883.77463

Computing standard errors:

Mixed-effects ML regression                     Number of obs     =        290
Group variable: gpnumberreal                    Number of groups  =         44

                                                Obs per group:
                                                              min =          1
                                                              avg =        6.6
                                                              max =         15

                                                Wald chi2(7)      =      21.97
Log likelihood = -883.77463                     Prob > chi2       =     0.0026

------------------------------------------------------------------------------------------
               deltasdq |      Coef.   Std. Err.      z    P>|z|     [95% Conf. Interval]
------------------------+-----------------------------------------------------------------
           sdqtotprobs0 |  -.2431686   .0777002    -3.13   0.002    -.3954582   -.0908791
             1.groupbin |   1.972318   1.131128     1.74   0.081    -.2446519    4.189287
       1.gpseesmorekids |   1.070316   1.219479     0.88   0.380    -1.319819     3.46045
                        |
groupbin#gpseesmorekids |
                   1 1  |  -4.599914   1.770463    -2.60   0.009    -8.069957    -1.12987
                        |
                wavebin |  -.9594846   .5858328    -1.64   0.101    -2.107696    .1887267
             publicsite |   .8376465   1.041367     0.80   0.421    -1.203395    2.878688
               caseload |   1.153832   .8444431     1.37   0.172    -.5012461     2.80891
                  _cons |  -.0444804   1.986436    -0.02   0.982    -3.937824    3.848863
------------------------------------------------------------------------------------------

------------------------------------------------------------------------------
  Random-effects Parameters  |   Estimate   Std. Err.     [95% Conf. Interval]
-----------------------------+------------------------------------------------
gpnumberreal: Identity       |
                  var(_cons) |   3.111449   1.501722       1.20819    8.012905
-----------------------------+------------------------------------------------
               var(Residual) |   23.74911   2.121588      19.93456    28.29359
------------------------------------------------------------------------------
LR test vs. linear model: chibar2(01) = 9.08          Prob >= chibar2 = 0.0013
```

Sensitivity analysis 2. Mixed analysis with time modeled as continuous variable ln(t+1) (no covariates)
Sdqtotprobs=SDQ total problems score

Groupbin=intervention (1) versus control (0)
Lntime=ln(time point coded as 0,1,2 +1)

```
. mixed sdqtotprobs i.groupbin##c.lntime || gpnumberreal: || Code:, mle

Performing EM optimization:

Performing gradient-based optimization:

Iteration 0:   log likelihood = -3030.8696
Iteration 1:   log likelihood = -3030.8695

Computing standard errors:

Mixed-effects ML regression                     Number of obs     =      1,017

-------------------------------------------------------------
                |     No. of       Observations per Group
 Group Variable |     Groups    Minimum    Average    Maximum
----------------+--------------------------------------------
   gpnumberreal |         49          1       20.8         51
```

© 2022 Sharifi V et al. *JAMA Psychiatry.*

```
           Code |        389          1        2.6          3
-------------------------------------------------------------

                                                Wald chi2(3)      =     216.25
Log likelihood = -3030.8695                     Prob > chi2       =     0.0000

------------------------------------------------------------------------------
     sdqtotprobs |      Coef.   Std. Err.      z    P>|z|     [95% Conf. Interval]
-----------------+------------------------------------------------------------
      1.groupbin |   .0476524   .7837756     0.06   0.952     -1.48852    1.583824
          lntime |   -4.25903   .3986458   -10.68   0.000    -5.040362   -3.477699
                 |
groupbin#c.lntime |
               1 |   .5987109   .5391645     1.11   0.267     -.458032    1.655454
                 |
           _cons |   20.00472   .5753548    34.77   0.000     18.87705     21.1324
------------------------------------------------------------------------------

------------------------------------------------------------------------------
  Random-effects Parameters  |   Estimate   Std. Err.     [95% Conf. Interval]
-----------------------------+------------------------------------------------
gpnumberreal: Identity       |
                  var(_cons) |    3.81338   1.263563      1.991886    7.300551
-----------------------------+------------------------------------------------
Code: Identity               |
                  var(_cons) |   10.37221   1.252109      8.186838    13.14094
-----------------------------+------------------------------------------------
               var(Residual) |   14.61284    .812327      13.10437    16.29494
------------------------------------------------------------------------------
LR test vs. linear model: chi2(2) = 257.53                Prob > chi2 = 0.0000

Note: LR test is conservative and provided only for reference.
```

Sensitivity analysis 3. Main effect and subgroup analysis using *average change* in SDQ score per GP

a. main effect

Deltasdq=*average* post-pre change in SDQ total problems score per GP
Groupbin=treatment versus control
Numpts=number of patients seen by each GP
Gpseesmorekids=child versus adult-predominant practice

```
. regress deltasdq i.groupbin [fweight=numpts]

      Source |       SS           df       MS      Number of obs   =       380
-------------+----------------------------------   F(1, 378)       =      1.69
       Model |  14.8710341         1  14.8710341   Prob > F        =    0.1942
    Residual |  3323.04896       378  8.79113482   R-squared       =    0.0045
-------------+----------------------------------   Adj R-squared   =    0.0018
       Total |     3337.92       379  8.80717677   Root MSE        =     2.965

------------------------------------------------------------------------------
    deltasdq |      Coef.   Std. Err.      t    P>|t|     [95% Conf. Interval]
-------------+----------------------------------------------------------------
  1.groupbin |   .3972411    .305426     1.30   0.194    -.2033058     .997788
       _cons |  -4.584096   .2254236   -20.34   0.000    -5.027337   -4.140855
------------------------------------------------------------------------------
```

b. subgroup analysis

```
. regress deltasdq i.groupbin##i.gpseesmorekids [fweight=numpts]

      Source |       SS           df       MS      Number of obs   =       370
-------------+----------------------------------   F(3, 366)       =     30.71
       Model |  667.187941         3   222.39598   Prob > F        =    0.0000
    Residual |  2650.15927       366  7.24087231   R-squared       =    0.2011
```

© 2022 Sharifi V et al. *JAMA Psychiatry.*

```
-------------+----------------------------------   Adj R-squared   =    0.1946
       Total |  3317.34721       369  8.99010084   Root MSE        =    2.6909

------------------------------------------------------------------------------------------
               deltasdq |      Coef.   Std. Err.      t    P>|t|     [95% Conf. Interval]
------------------------+-----------------------------------------------------------------
             1.groupbin |   2.386845   .3792573     6.29   0.000     1.641048    3.132642
       1.gpseesmorekids |   1.721008   .4099983     4.20   0.000     .9147598    2.527256
                        |
groupbin#gpseesmorekids |
                   1 1  |   -5.22099   .5793789    -9.01   0.000    -6.360319    -4.08166
                        |
                  _cons |  -5.499314   .2989874   -18.39   0.000    -6.087263   -4.911365
------------------------------------------------------------------------------------------
```

Sensitivity analysis 4. Main effect and subgroup analysis using *average SDQ score per GP* in multilevel model

Variables as above except sdqgpavg= the average SDQ total problems score among all children seeing the GP at each time point

a. main effect

```
. mixed sdqgpavg i.groupbin##i.timepoint || gpnumberreal:, mle

Performing EM optimization:

Performing gradient-based optimization:

Iteration 0:   log likelihood = -348.09532
Iteration 1:   log likelihood = -348.09532

Computing standard errors:

Mixed-effects ML regression                     Number of obs     =        141
Group variable: gpnumberreal                    Number of groups  =         49

                                                Obs per group:
                                                              min =          1
                                                              avg =        2.9
                                                              max =          3

                                                Wald chi2(5)      =      95.05
Log likelihood = -348.09532                     Prob > chi2       =     0.0000

-------------------------------------------------------------------------------------
          sdqgpavg |      Coef.   Std. Err.      z    P>|z|     [95% Conf. Interval]
-------------------+-----------------------------------------------------------------
        1.groupbin |  -.3501693   .9773543    -0.36   0.720    -2.265749     1.56541
                   |
         timepoint |
                1  |  -3.148503   .6366432    -4.95   0.000    -4.396301   -1.900705
                2  |  -4.104382   .6268899    -6.55   0.000    -5.333064   -2.875701
                   |
groupbin#timepoint |
              1 1  |   .7222442   .8810876     0.82   0.412    -1.004656    2.449144
              1 2  |  -.1679714   .8805046    -0.19   0.849    -1.893729    1.557786
                   |
             _cons |   20.13734   .7119355    28.29   0.000     18.74197     21.5327
-------------------------------------------------------------------------------------

------------------------------------------------------------------------------
  Random-effects Parameters  |   Estimate   Std. Err.     [95% Conf. Interval]
-----------------------------+------------------------------------------------
gpnumberreal: Identity       |
                  var(_cons) |   7.138205   1.783291      4.374605    11.64767
-----------------------------+------------------------------------------------
               var(Residual) |   4.519396   .6657029      3.386095    6.032004
```

© 2022 Sharifi V et al. *JAMA Psychiatry.*

```
------------------------------------------------------------------------------
LR test vs. linear model: chibar2(01) = 50.38         Prob >= chibar2 = 0.0000
```

b. subgroup analysis

```
mixed sdqgpavg i.groupbin##i.timepoint##i.gpseesmorekids|| gpnumberreal:, mle

Performing EM optimization:

Performing gradient-based optimization:

Iteration 0:   log likelihood = -343.52685
Iteration 1:   log likelihood = -343.52685

Computing standard errors:

Mixed-effects ML regression                     Number of obs     =        141
Group variable: gpnumberreal                    Number of groups  =         49

                                                Obs per group:
                                                              min =          1
                                                              avg =        2.9
                                                              max =          3

                                                Wald chi2(11)     =     108.11
Log likelihood = -343.52685                     Prob > chi2       =     0.0000

-----------------------------------------------------------------------------------------------
                     sdqgpavg |      Coef.   Std. Err.      z    P>|z|     [95% Conf. Interval]
------------------------------+----------------------------------------------------------------
                   1.groupbin |  -.4529662   1.212161    -0.37   0.709    -2.828758    1.922826
                              |
                    timepoint |
                           1  |  -2.491816   .8521816    -2.92   0.003    -4.162061   -.8215703
                           2  |  -3.866568   .8521816    -4.54   0.000    -5.536813   -2.196322
                              |
           groupbin#timepoint |
                         1 1  |   .3047294   1.110392     0.27   0.784    -1.871599    2.481057
                         1 2  |   .0446524   1.120233     0.04   0.968    -2.150964    2.240269
                              |
             1.gpseesmorekids |  -1.308682   1.357699    -0.96   0.335    -3.969723    1.352359
                              |
      groupbin#gpseesmorekids |
                         1 1  |  -.3913752    1.93739    -0.20   0.840     -4.18859     3.40584
                              |
     timepoint#gpseesmorekids |
                         1 1  |  -1.446982   1.254084    -1.15   0.249    -3.904942    1.010978
                         2 1  |  -.4972486   1.232253    -0.40   0.687    -2.912419    1.917922
                              |
groupbin#timepoint#gpseesmorekids |
                       1 1 1  |   .6042997    1.81449     0.33   0.739    -2.952035    4.160635
                       1 2 1  |  -1.005248   1.805559    -0.56   0.578    -4.544079    2.533584
                              |
                        _cons |   20.76323   .9389358    22.11   0.000     18.92295    22.60351
-----------------------------------------------------------------------------------------------

------------------------------------------------------------------------------
  Random-effects Parameters  |   Estimate   Std. Err.     [95% Conf. Interval]
-----------------------------+------------------------------------------------
gpnumberreal: Identity       |
                  var(_cons) |   6.221925   1.579957      3.782474    10.23466
-----------------------------+------------------------------------------------
               var(Residual) |   4.357281   .6405561      3.266495    5.812316
------------------------------------------------------------------------------
LR test vs. linear model: chibar2(01) = 46.50         Prob >= chibar2 = 0.0000

. marginsplot
  Variables that uniquely identify margins: timepoint groupbin gpseesmorekids
.
```

© 2022 Sharifi V et al. *JAMA Psychiatry.*

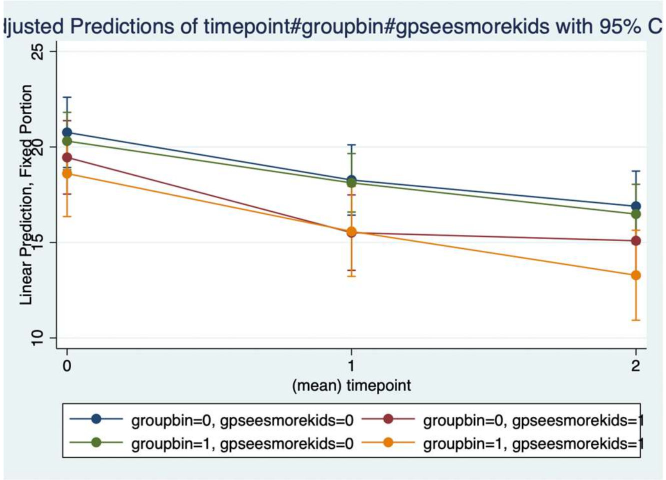

© 2022 Sharifi V et al. *JAMA Psychiatry.*
