# Supplementary material for: Effect of General Practitioner Training in a Collaborative Child Mental Health Care Program on Children’s Mental Health Outcomes in a Low-Resource Setting: A Cluster Randomized Trial
Source: JAMA Psychiatry. 2022 Nov 30;80(1):22–30. doi: 10.1001/jamapsychiatry.2022.3989 (PMC9713683; doi:10.1001/jamapsychiatry.2022.3989)
Supplement: Supplement 3. — Data Sharing Statement [file jamapsychiatry-e223989-s003.pdf]

Data Sharing Statement

Sharifi. Effect of General Practitioner Training in a Collaborative Child Mental Health Care Program on Children's Mental Health Outcomes in a Low-Resource Setting. *JAMA Psychiatry*. Published November 30, 2022. doi:10.1001/jamapsychiatry.2022.3989

Data
Data available: Yes
Data types: Deidentified participant data
How to access data: US National Database for clinical trials related to mental illness
When available: With publication

Supporting Documents
Document types: Other (please specify)
Additional Information: Published trial protocol
How to access documents: Sharifi V, Shahrivar Z, Zarafshan H, Ashkezary SB, Stuart E, Mojtabai R, Wissow L. Collaborative care for child and youth mental health problems in a middle-income country: study protocol for a randomized controlled trial training general practitioners. Trials. 2019 Jul 8;20(1):405. doi: 10.1186/s13063-019-3467-4. PMID: 31287011; PMCID: PMC6615304.
When available: With publication

Additional Information
Who can access the data: Anyone
Types of analyses: Any purpose related to child mental health research
Mechanisms of data availability: Per terms of the US National Database for clinical trials related to mental illness
Any additional restrictions: None
